# Association of Red Meat Consumption, Metabolic Markers, and Risk of Cardiovascular Diseases

**DOI:** 10.3389/fnut.2022.833271

**Published:** 2022-04-15

**Authors:** Lang Pan, Lu Chen, Jun Lv, Yuanjie Pang, Yu Guo, Pei Pei, Huaidong Du, Ling Yang, Iona Y. Millwood, Robin G. Walters, Yiping Chen, Yujie Hua, Rajani Sohoni, Sam Sansome, Junshi Chen, Canqing Yu, Zhengming Chen, Liming Li

**Affiliations:** ^1^Department of Epidemiology and Biostatistics, School of Public Health, Peking University, Beijing, China; ^2^Center for Public Health and Epidemic Preparedness and Response, Peking University, Beijing, China; ^3^Key Laboratory of Molecular Cardiovascular Sciences, Ministry of Education, Peking University, Beijing, China; ^4^Fuwai Hospital Chinese Academy of Medical Sciences, National Center for Cardiovascular Diseases, Beijing, China; ^5^National Center for Cardiovascular Diseases, Chinese Academy of Medical Sciences, Beijing, China; ^6^Medical Research Council Population Health Research Unit, University of Oxford, Oxford, United Kingdom; ^7^Clinical Trial Service Unit and Epidemiological Studies Unit, Nuffield Department of Population Health, University of Oxford, Oxford, United Kingdom; ^8^Noncommunicable Diseases Prevention and Control Department, Suzhou Center for Disease Control and Prevention, Suzhou, China; ^9^China National Center for Food Safety Risk Assessment, Beijing, China

**Keywords:** red meat, processed meat, metabolomics, lipoproteins, cardiovascular diseases

## Abstract

**Objective:**

The metabolic mechanism of harmful effects of red meat on the cardiovascular system is still unclear. The objective of the present study is to investigate the associations of self-reported red meat consumption with plasma metabolic markers, and of these markers with the risk of cardiovascular diseases (CVD).

**Methods:**

Plasma samples of 4,778 participants (3,401 CVD cases and 1,377 controls) aged 30–79 selected from a nested case-control study based on the China Kadoorie Biobank were analyzed by using targeted nuclear magnetic resonance to quantify 225 metabolites or derived traits. Linear regression was conducted to evaluate the effects of self-reported red meat consumption on metabolic markers, which were further compared with the effects of these markers on CVD risk assessed by logistic regression.

**Results:**

Out of 225 metabolites, 46 were associated with red meat consumption. Positive associations were observed for intermediate-density lipoprotein (IDL), small high-density lipoprotein (HDL), and all sizes of low-density lipoprotein (LDL). Cholesterols, phospholipids, and apolipoproteins within various lipoproteins, as well as fatty acids, total choline, and total phosphoglycerides, were also positively associated with red meat consumption. Meanwhile, 29 out of 46 markers were associated with CVD risk. In general, the associations of metabolic markers with red meat consumption and of metabolic markers with CVD risk showed consistent direction.

**Conclusions:**

In the Chinese population, red meat consumption is associated with several metabolic markers, which may partially explain the harmful effect of red meat consumption on CVD.

## Introduction

Cardiovascular disease (CVD) is an important part of noncommunicable disease (NCD) as well as a major public health concern, resulting in 18.6 million deaths and 34.4 million years lived with disability (YLDs) worldwide in 2019 (1, 2). Ischemic heart disease (IHD), ischemic stroke (IS), and intracerebral hemorrhage (ICH) accounted for the majority of the deaths caused by CVD, which become even more severe in developing countries with aging populations, including China (1, 3). In 2017, cerebrovascular disease and IHD ranked the top two leading causes of death in the Chinese population (2, 4–7).

Red meat is named after the color of myoglobin. Compared with poultry, fish, and other white meat, red meat such as pork, beef, and mutton contains higher triglycerides and saturated fatty acids. The harmful effect of red meat on health has been widely proved. Evidence from cohort studies suggested that unprocessed and processed red meat were positively associated with risks of coronary heart disease (CHD) and all-cause mortality (8, 9). A meta-analysis involving 61 studies and more than 4 million participants found that 3 servings per week reduction in unprocessed red meat intake was associated with lower risks of myocardial infarction (MI), stroke, and type 2 diabetes (T2D) (10). Similar associations were reported in two other large meta-analyses (11, 12).

Closely linked with red meat consumption, the disorder of plasma lipids metabolism, especially low-density lipoprotein cholesterol (LDL-C), is generally considered one of the biological mechanisms behind IHD and stroke risks, by accumulating in the arterial wall, gradually forming atherosclerotic plaques, and eventually blocking the corresponding artery (5–7). Beyond traditional biochemical approaches, lipoproteins could be further categorized into large, medium, and small particle sizes and comprise a series of subfractions, including cholesterol, phospholipids, triglycerides, and apolipoprotein. Nuclear magnetic resonance (NMR) metabolomics is an emerging field with the potential to quantify small-molecule metabolic markers in a more detailed perspective (13).

However, no study based on the same population has yet simultaneously assessed the associations of red meat consumption with individual plasma metabolites and of these metabolites with CVD risk, which may provide more homogeneous insights into the biological mechanisms linking red meat consumption and CVD risk. Furthermore, dietary guidelines have treated limiting red meat consumption as the primary recommendation in many countries, including China (14, 15). With the increasing trend of red meat consumption in China, (16) there is an increasing need to assess the role of individual metabolites in the association between red meat and CVD risk in the same Chinese population, to provide more evidence from a metabolomics perspective.

The present study aimed at simultaneously exploring the associations of self-reported red meat consumption with plasma lipids, fatty acids, amino acids, and other metabolic markers, and of these markers with risk of CVD in a nested case-control study in China Kadoorie Biobank (CKB).

## Materials and Methods

### Participants and Study Design

CKB is a prospective cohort of 5,12,725 adults recruited between 2004 and 2008 from 5 urban and 5 rural areas across China, whose morbidity and mortality were followed up once enrollment. The study design and methods of CKB have been described in detail elsewhere (17). Briefly, after signing a written informed consent form, baseline information was collected using a laptop-based questionnaire, such as demographic characteristics (e.g., age, sex, education, household income, and marital status), lifestyle factors (e.g., drinking and smoking habits, food intake, and physical activities), medical history (e.g., hypertension, diabetes, and use of certain specific medications such as statins), and family history of diabetes, heart attack or stroke. All participants also provided a 10mL non-fasting (with time since last meal recorded) blood sample for a quick on-site test of random plasma glucose (RPG) and long-term storage. In addition, every 5 years after completing the baseline survey, about 5% of the participants were randomly selected to join in the re-survey.

The present study included 4,778 participants, who had metabolomics data, from a previous nested case-control study based on CKB (18). Cases consisted of incident cases of MI (ICD-10 I21-23, *n* = 946), IS (I63 and I69.3, *n* = 1,217), and ICH (I61 and I69.1, *n* = 1,238), with a censoring date of 1 January 2015. And 1,377 controls were frequently matched to the combined cases by age, sex, and area if possible. All cases and controls had no history of self-reported prior doctor-diagnosed coronary heart disease (CHD), stroke, transient ischemic attack, and cancer, and were not using statin therapy at baseline. The Ethical Review Committee of the Chinese Center for Disease Control and Prevention (Beijing, China) and the Oxford Tropical Research Ethics Committee, University of Oxford (UK) approved the study.

### Assessment of Red Meat Consumption

Using the interviewer-administered, laptop-based food frequency questionnaire (FFQ) at baseline, participants were asked about their habitual frequency of 12 food groups during the past 12 months, including rice, wheat, other staples (such as corn and millet), red meat, poultry, fish, eggs, fresh fruit, fresh vegetables, preserved vegetables, soybean, and dairy products. Possible answers were “never/rarely, monthly, 1–3 days per week, 4–6 days per week, and daily”. The frequency was then converted into weekly days of red meat consumption, with each option corresponding to 0, 0.5, 2, 5, and 7 days per week, respectively. In the 2nd re-survey (2013–2014), 23,974 participants were additionally asked about their daily amount when consuming red meat. A validated picture booklet was used to assess portion sizes. The average daily consuming amount according to sex, age group (<60y or ≥60y), region (10 regions), and frequency group (never/rarely, monthly, 1–3 d/w, 4–6 d/w, or daily) was calculated as a proxy to approximate the amount of red meat consumption.

In order to evaluate the reproducibility and validity of FFQs used in baseline and re-surveys, a separate validation study, whose detailed protocol and results have been described elsewhere, (19) was conducted from 2015 to 2016 among 432 CKB participants according to sex, age group, and regions in four survey sites of the whole CKB study. These participants completed two FFQs (median interval: 3.3 months) and twelve 24-h dietary recalls (24-HDR) with 3 weekdays and 1 weekend day in each season except spring. The weighted Kappa statistic was 0.77 for reproducibility of baseline red meat frequency and 0.62 for red meat consumption amount in 2nd re-survey. Compared with the average of twelve 24-HDRs used as the “gold standard”, the weighted Kappa statistic was 0.73 for relative validity of baseline food frequency and 0.68 for relative validity of red meat consumption amount in 2nd re-survey. The FFQs used at baseline and in the 2nd re-survey, as well as the 24 h dietary recall table were shown in the Supplementary Materials.

### Measurement of NMR Metabolomics

After centrifuging and aliquoting, baseline plasma samples were couriered to Oxford for long-term storage in liquid nitrogen tanks. The stored plasma samples of the cases and controls were thawed and sub-aliquoted at the Wolfson laboratory, CTSU, before 100uL aliquots being shipped on dry ice to the Brainshake Laboratory at Oulu, Finland, for high-throughput targeted NMR spectroscopy to quantify 225 absolute concentrations of metabolic markers or derived traits (e.g., lipids ratios) (20). The samples were handled in 96-well plates, every plate containing 2 quality control samples (a plasma mimic and a mixture of 2 low-molecular-weight metabolites). The former was used to monitor the consistency of quantifications, whereas the latter was a technical reference to monitor the performance of the automated liquid handler and the spectrometer. Barcoding was preferred for sample identification. Before the NMR measurements, 260 μL of plasma and 260 μL of sodium phosphate buffer were carefully mixed and moved to the NMR tubes. All the liquid handling steps for native plasma samples were done with an automated workstation equipped with an 8-tip dispense arm.

The NMR metabolomics was based on three molecular windows. Lipoprotein lipids (LIPO) and low-molecular-weight metabolites (LMWM) were applied to native plasma, while lipid extracts (LIPID) were applied after a standardized lipid extraction procedure based on multiple steps to break down the lipoprotein particles, containing saturated sodium chloride solution, methanol, dichloromethane, deuterochloroform, as well as centrifugation and evaporation (21). The laboratory setup successively used Bruker AVANCE III 500 MHz (LIPO and LMWM) and Bruker AVANCE III HD 600 MHz (LIPID) spectrometer. The LIPO data were recorded after four dummy scans using 8 transients acquired with an automatically calibrated 90° pulse and applying a noesypresat pulse sequence with a mixing time of 10 ms and irradiation field of 25 Hz to suppress the water peak. The acquisition time was 2.7 s, and the relaxation delay was 3.0 s. The 90° pulse was calibrated automatically for each sample. The LMWM data were acquired using 24 transients after four steady-state scans with a Bruker 1D CPMG pulse sequence. The LIPID data were obtained using 32 transients after four dummy scans. For LMWM and LIPID data, a relaxation delay of 3.0 s and an acquisition time of 3.3 s were used. The free induction decays (FIDs) were zero-filled to 128 k data points and then multiplied with an exponential window function with a 1.0 Hz line broadening for LIPO and LMWM and 0.5 Hz for LIPID. Each LIPID spectrum was scaled according to the total cholesterol estimated from the corresponding LIPO spectrum using a regression model.

The initial data processing, including the Fourier transformations to NMR spectra and automated phasing, were done using the computers that control the spectrometers. The spectra were then automatically transferred to a centralized server, which performed various further automatic spectral processing steps, including an overall signal check for missing/extra peaks, background control, baseline removal, and spectral area-specific signal alignments. The spectral information of the actual sample also underwent various comparisons with the spectra of the 2 quality control samples. Regression modeling was performed to produce the quantified molecular data for those spectral areas that passed all the quality control steps. Also, the individual metabolic measures underwent various statistical quality control steps and were also checked against an extensive database of quantitative molecular data. The metabolic measures that passed all quality control steps were stored in the database. Samples from cases and controls were quantified randomly, with laboratory staff blinded to case or control status. The sample size of some metabolic markers involved was <4,778 since the quality control process rejected results of these metabolic markers among some participants.

### Follow-Up for Incident CVD

The incident cases of MI, IS, and ICH were identified through electronic linkage via a unique personal identification number to established death registries and the universal nationwide health insurance system, which provides coded disease diagnoses and procedures for each hospitalization of participants on a 6-monthly basis.

### Statistical Analysis

Baseline characteristics were presented as means or percentages across controls and 3 subtypes of CVD cases, using multiple linear regressions for continuous variables or logistic regressions for categorical variables.

For each metabolite, measurements below the limit of detection were imputed with the lowest measured concentration. Each metabolic marker was log-transformed and divided by its standard deviation (SD). Linear regression was used to assess the associations of red meat consumption with metabolic markers, adjusted for age, sex, region, education, household income, occupation, marital status, tea-drinking habit, smoking status, alcohol intake, physical activity, self-rated health, fasting time, and frequency of other 11 food groups. For each biomarker, adjusted SD differences of log-transformed metabolic markers and 95% confidence intervals (CI) associated with an extra weekly day of red meat consumption were estimated.

Logistic regression was used to estimate odds ratios (ORs) for CVD and its 3 subtypes (MI, IS, and ICH) per SD higher log-transformed metabolic markers, with the same variables adjusted for as in the analysis of red meat consumption and metabolic markers. ICH cases were excluded from the analysis of metabolic markers and CVD because there were no associations of metabolic markers with ICH (18). ORs were then plotted against SD differences in corresponding log-transformed metabolic biomarkers per extra day of red meat consumption. A flowchart that detailed the data analysis and integration was shown in Supplementary Figure S1.

We performed several sensitivity analyses to examine the robustness of associations between red meat consumption and metabolic markers, including additionally adjusting for body mass index (BMI), presence of prevalent hypertension or diabetes, and family history of diabetes or CVD. We also re-run the analyses when excluding participants with any metabolic markers below the limit of detection or rejected by quality control, and using the weekly amount of red meat consumption as a continuous independent variable instead of its frequency.

All *p-*values were two-sided, and statistical significance was defined as *p* < 0.05. To account for a large number of highly correlated metabolic markers, we calculated the false discovery rate (FDR) *p* < 0.05 based on the Benjamini-Hochberg method for associations of red meat consumption with metabolic markers and of metabolic markers with risk of CVD. Statistical analyses were performed using Stata 15.0.

## Results

Baseline characteristics of the 4,778 participants according to whether they developed CVD are shown in Table 1. Briefly, the mean (SD) age was 47.0 (8.2) years, 50.1% were women, and 29.0% resided in urban areas. The mean (SD) frequency of red meat consumption and plasma total cholesterol concentration were 3.3 (2.6) days per week and 3.5 (0.6) mmol/L, respectively. The mean values and SDs of all 225 metabolites were shown in Supplementary Table S1. Compared with controls, participants who subsequently developed any subtype of CVD were more likely to report poor self-rated health, prevalent diabetes, hypertension, and a family history of CVD. Among them, MI or IS cases had higher educational attainment and annual household income, and were more likely to be regular smokers. Whereas, ICH cases had lower educational attainment but higher levels of SBP and DBP.

**Table 1:** Baseline characteristics among 4,778 participants.

	**Controls**	**CVD cases**		
		**All cases**	**MI or IS cases**	**ICH cases**
*N*	1,377	3,401	2,163	1,238
Age, y	46.95 (9.00)	47.01 (7.88)	46.76 (8.35)	47.44 (6.99)
Female, %	50.47	49.93	48.45	52.5
Urban residents, %	27.09	29.76	33.56	23.1
Middle school or above, %	55.7	55.28	57.47	51.45
Income ≥35,000 RMB/year, %	10.02	12.06	13.08	10.26
Manual worker, %	70.88	69.36	67.04	73.42
BMI, kg/m^2^	23.53 (3.27)	24.19 (3.64)	24.28 (3.67)	24.04 (3.57)
SBP, mmHg	127.85 (18.26)	141.48 (27.21)	136.76 (24.88)	149.73 (29.09)
DBP, mmHg	76.89 (10.56)	84.93 (15.16)	82.59 (14.03)	89.01 (16.16)
Ever regular smoking, %	34.42	38.40	40.45	34.81
Weekly drinking, %	18.01	18.38	17.85	19.31
Physical activity, MET h/d	23.36 (14.29)	22.67 (14.44)	22.37 (14.32)	23.20 (14.63)
Red meat consumption, d/w	3.26 (2.53)	3.31 (2.61)	3.36 (2.65)	3.21 (2.55)
Fresh vegetables consumption, d/w	6.81 (0.82)	6.77 (0.96)	6.77 (0.97)	6.78 (0.94)
Fresh fruit consumption, d/w	2.29 (2.37)	2.03 (2.24)	2.10 (2.33)	1.91 (2.08)
≥8 h of fasting, %	13.65	15.64	17.06	13.17
Poor self-rated health, %	9.73	13.79	13.82	13.73
Diabetes, %	5.45	9.14	9.75	8.08
Hypertension, %	26.94	52.19	45.21	64.38
Family history of diabetes, %	6.9	8.88	9.99	6.95
Family history of CVD, %	23.02	28.76	28.43	29.32

*Values are means (standard errors, SE) or %*.

**Figure 1:**
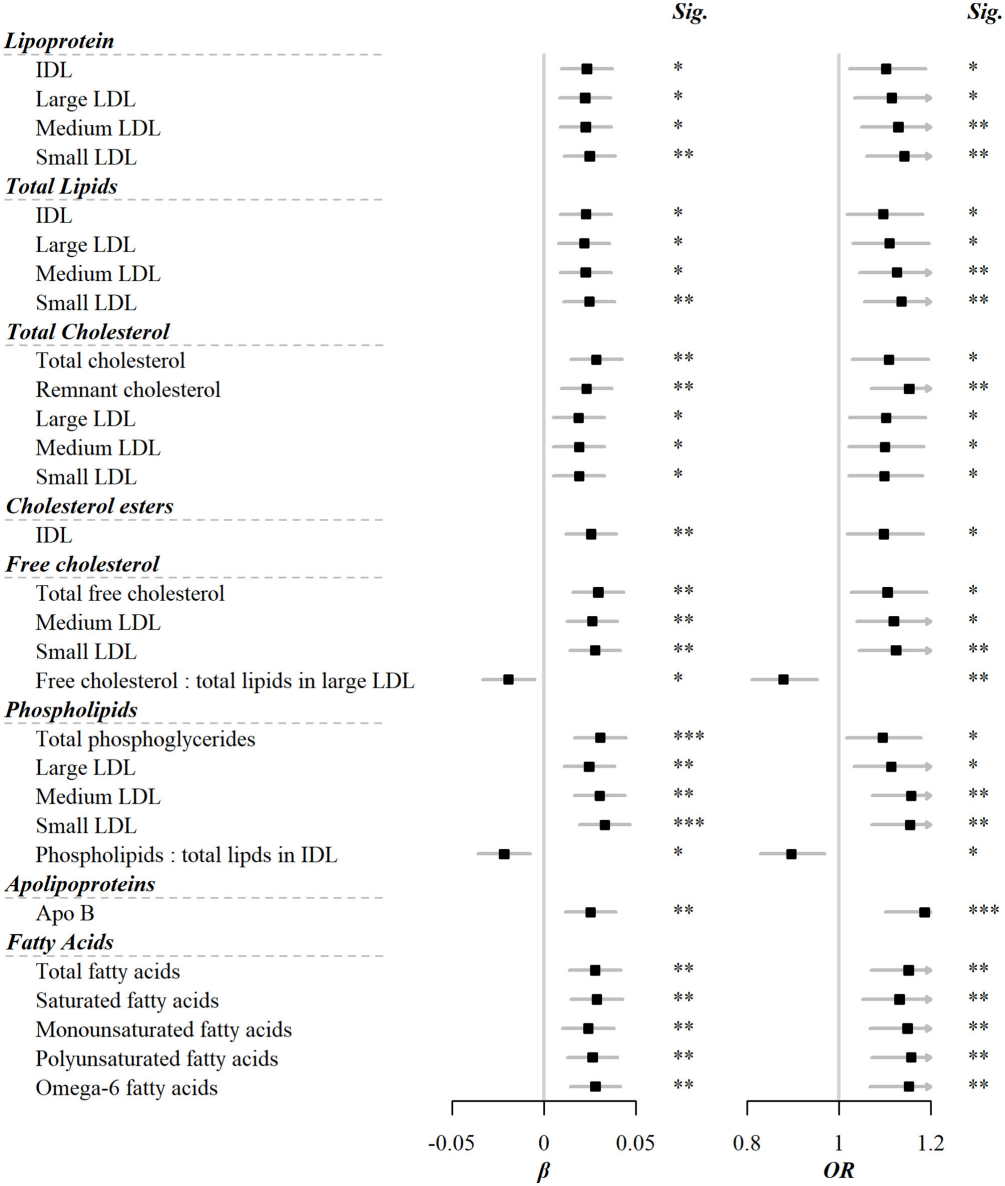
Metabolic markers that significantly associated with both red meat consumption and risks of CVD. Models were adjusted for age, sex, region, education, household income, occupation, marital status, tea-drinking habit, smoking status, alcohol intake, physical activity, self-rated health, fasting time, and frequency of other 11 food groups. Black squares represented coefficients or ORs, while gray horizontal lines represented 95%CI. Significance (Sig.): *p < 0.05, **p < 0.01, ***p < 0.001 (FDR-adjusted *p* using the Benjamini-Hochberg method).

Among the 225 metabolic markers or derived traits, 46 were associated with the frequency of red meat consumption at FDR <5% (Figure 1), the majority of which showed a significant increase. Red meat consumption was positively associated with concentrations of IDL and all particle sizes of LDL. Similar associations were also shown for total lipids, cholesterols, and phospholipids in these particles. Besides, total cholesterol, total free cholesterol, total fatty acids, total choline, and total phosphoglycerides were also positively associated with red meat consumption. It should be pointed out that, although both saturated and unsaturated fatty acids were positively associated with red meat intake, the ratio of 22:6 docosahexaenoic acid (DHA, an essential dietary polyunsaturated fatty acid) to total fatty acids showed a negative association. The associations for all 225 markers are shown in Supplementary Table S2.

**Figure 2:**
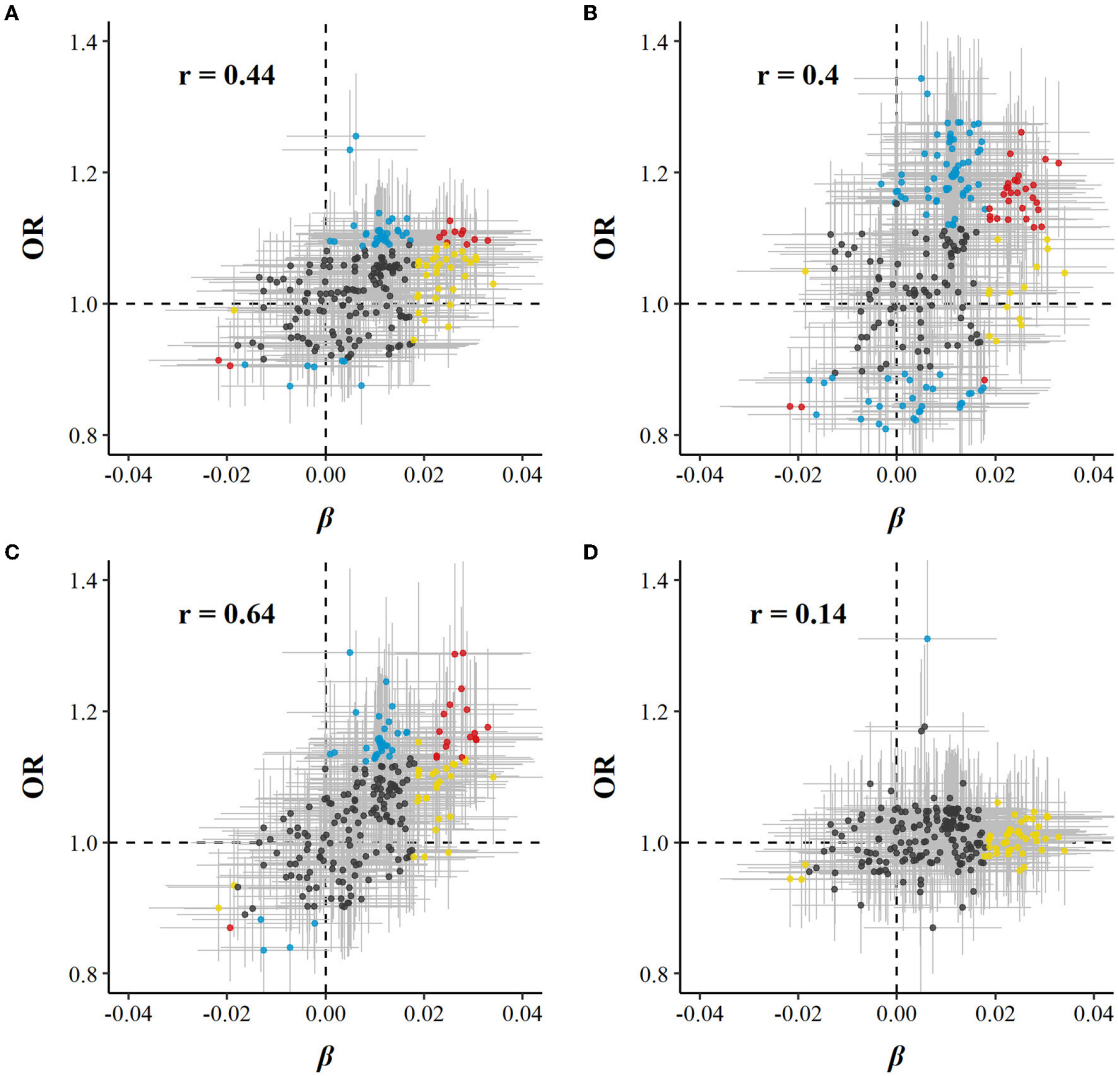
Global comparison of SD differences of 225 log-transformed metabolic markers associated with red meat consumption vs. ORs for **(A)** CVD, **(B)** MI, **(C)** IS, and **(D)** ICH associated with SD higher log-transformed metabolic markers. Models were adjusted for age, sex, region, education, household income, occupation, marital status, tea-drinking habit, smoking status, alcohol intake, physical activity, self-rated health, fasting time, and frequency of other 11 food groups. Yellow dots represented markers that associated with red meat consumption but not with risk of diseases; blue dots represented markers that associated with risk of diseases but not with red meat consumption; and red dots represented markers that associated with both red meat consumption and risk of diseases, with overlapping dots darker in color. The gray horizontal line and vertical line represented 95%CI of coefficients and ORs, respectively. Pearson correlations of coefficients and ORs were annotated in the upper left corner.

Of 225 metabolic markers or derived traits, 104, 123, 55, and 1 were associated with CVD and its subtypes, MI, IS, and ICH, respectively, at FDR <5% (Supplementary Tables S2, S3). For saturated fatty acids, each SD increment of log-transformed value was associated with increased risks of CVD [OR 1.13 (95%CI 1.05–1.22)], MI [1.14 (1.04–1.25)], and IS [1.20 (1.09–1.32)], whereas the association with total cholesterol within HDL was in the opposite direction [0.91 (0.85–0.98) for CVD and 0.86 (0.79–0.95) for MI]. ORs for MI, IS, and ICH associated with all 225 metabolic markers are provided in Supplementary Table S3. Among markers associated with risk of CVD, MI, or IS, 29, 31, and 18 were simultaneously associated with red meat consumption. There was consistency in the direction between the associations of red meat consumption with metabolites and these metabolites with risks of CVD, MI, and IS. In other words, when red meat consumption was associated with altered levels of metabolites, the corresponding altered levels of that same metabolites were associated with a higher risk of CVD, MI, and IS (Pearson correlation: 0.44, 0.40, and 0.64, respectively; Supplementary Table S2, Figure 2).

In sensitivity analyses, associations between red meat consumption and metabolic markers persisted when BMI, hypertension, diabetes, and family history of diabetes or CVD were added to the basic model (Supplementary Figure S2). Similar associations were observed when restricting the analyses to participants without any metabolic markers below the limit of detection or rejected by the quality control (*n* = 4,251), and using the weekly amount of red meat consumption as a continuous independent variable instead of its frequency (Supplementary Figure S3).

## Discussion

In the current study based on data of NMR metabolomics in a Chinese population, our study showed that red meat consumption was positively associated with concentrations of total lipids, cholesterols, and phospholipids within IDL and all particle sizes of LDL, as well as total cholesterol, free cholesterol, total fatty acids, total choline, and total phosphoglycerides. Moreover, most of these metabolic markers were positively associated with the risk of CVD. In contrast, negative associations were observed between red meat consumption and the ratio of DHA to total fatty acids. Globally, 225 metabolic markers showed a directionally consistent association with red meat consumption and CVD risk. Our results potentially explained the positive correlation between red meat consumption and CVD risk in the Chinese population through a metabolomics sight.

To our knowledge, although a series of metabolomics studies focused on red meat consumption, few showed further interest in lipid-related metabolites (22–24). A cohort study included 1,008 white participants and found that habitual pork and processed meat intake was associated with total fecal cholesterol, but did not explore lipoprotein with different density, diameter, and chemical structure in detail (25).

Although most were small sample interventions, previous results obtained by traditional biochemical detection technology supported our study. A crossover intervention based on 17 postmenopausal women found that consuming higher-monounsaturated fatty acid (MUFA) contained ground beef for 6 weeks significantly increased plasma LDL-C (26). This association was found to be modified by active physical activity, which highlighted the reasonableness of additional adjustment for physical activity levels in our study. Another study showed that reducing red and processed meat reduced total, LDL, and HDL cholesterol in males (27). In addition, two trials have shown that, compared to animal-based meat consumers, plant-based meat or meat analogs consumers had lower levels of TC and LDL-C (28, 29). The above results were in line with our study.

Among the cardiovascular risk biomarkers in the present study that correlated to red meat consumption, LDL and its subfractions, including total cholesterols, free cholesterols, and phospholipids, were the majority. This potentially gave us a picture of the metabolism triggered by red meat consumption. LDL was the product of a series of VLDL metabolism, including enzymatic hydrolysis of triglycerides, transport of free cholesterols and phospholipids, and reception of cholesterol esters bound from extrahepatic tissues by HDL. Thus, LDL reflected both VLDL and HDL metabolism, and played the role of transporting cholesterol toward the liver for further transformation to bile acid. On the other hand, IDL was an intermediate metabolite from VLDL to LDL, and still contained a considerable amount of triglycerides that had not been hydrolyzed; residual cholesterol refers to cholesterol within triglyceride-rich VLDL and IDL particles (30). Residual cholesterol has been identified as a cardiovascular risk factor independent of HDL in previous studies (31). Our study had further identified it as a biomarker correlated to red meat consumption. The inference was that higher red meat consumption led to more triglycerides and cholesterol, which triggered higher triglyceride enzymatic hydrolysis and cholesterol transport, reflecting as elevated residual lipoproteins, LDL, and IDL levels in the present study.

For fatty acids, the present study found that no matter total, saturated, or unsaturated fatty acids, they were positively correlated with red meat consumption, and the association with incident CVD risk also went in the same direction. On the one hand, this might be because red meat, while rich in saturated fatty acids, still contains unsaturated fatty acids, and the proportion is highly dependent on the type of red meat. On the other hand, as previous studies have pointed out, increased total triglyceride consumption may confound the association between unsaturated fatty acids and CVD risk (18).

Notably, the present study could provide Chinese-population-based evidence for the restriction of red meat consumption in current dietary guidelines in China. According to the latest Dietary Guidelines for Chinese Residents published in 2016, each standard adult should limit the total intake of poultry and red meat to 40–75 grams per day (14). However, not surprisingly, according to the China Statistical Yearbook, the average total purchase of pork, beef, and mutton by Chinese residents in 2019 was 26.9 kilograms, about 73.7 grams per day (32). Therefore, more effective health promotion strategies should be recommended to reduce red meat consumption and improve the plasma metabolites profile and cardiovascular health.

This study has several strengths, such as relatively large sample size, accurately identified CVD and its subtype events, collection of as many covariates as possible, and the detection of a wide range of metabolic markers based on the NMR platform, including but not limited to lipoproteins and their constituents with different particle size, density, and chemical structure. Our study also had limitations. First of all, similar to other large-scale nutritional epidemiological studies, there was an unavoidable recall bias when estimating red meat and other food consumption by FFQ. Measurement bias also occurred once five options were used to estimate the days of red meat consumption per week. However, associations showed in our study were consistent with those previous observational studies or interventions, and persisted when the imputed amount of red meat consumption per week was used as the independent variable. Second, fasting time before blood drawing affected metabolomics characteristics, especially lipid metabolism, although we included fasting time as a covariable in the model. Moreover, blood draws in the morning or evening may display major-specific signatures. Further nutritional metabolomics studies focusing on fasting blood or blood drawing time of day will substantially complement the present study. Third, residual confounding due to uncollected or suboptimally collected factors still existed, even if potential confounding factors such as BMI, frequency of other food consumption, history of chronic diseases, and family history were adjusted in multivariate models or sensitivity analysis. Although we adjusted for covariates related to total energy intake, such as alcohol intake, tea-drinking habits, 11 other food groups, and physical activity levels, the absence of accurate total energy intake may still lead to confounding between red meat consumption amount and plasma biomarkers. Finally, due to the cross-sectional nature, the strength of the causal relationship between baseline red meat consumption and baseline levels of metabolic markers are not as strong as those in a longitudinal design, which would be exciting additions to the present study.

This study based on a Chinese population found associations of red meat consumption with fatty acids, lipid-related metabolites within IDL, and all sizes of LDL. These associations were directionally consistent with associations between these metabolites and CVD risk. These results we reported potentially reveal that lipid-related metabolites may play a role in the harmful effects of red meat consumption on CVD at the molecular level, and provide Chinese population-based evidence for formulating strategies and policies to reduce red meat consumption.

## Data Availability Statement

The original contributions presented in the study are included in the article/Supplementary Materials, further inquiries can be directed to the corresponding author.

## Ethics Statement

The studies involving human participants were reviewed and approved by the Ethical Review Committee of the Chinese Center for Disease Control and Prevention (Beijing, China); The Oxford Tropical Research Ethics Committee, University of Oxford (UK). The patients/participants provided their written informed consent to participate in this study.

## Author Contributions

LP: formal analysis, writing—original draft, and writing—review and editing. LC: validation and writing—review and editing. JL and HD: methodology and writing—review and editing. YP, LY, IM, RW, YC, and YH: writing—review and editing. YG and PP: methodology, investigation, and writing—review and editing. RS and SS: software and writing—review and editing. JC, ZC, and LL: conceptualization, methodology, and writing—review and editing. CY: data curation and writing—review and editing. All authors contributed to the article and approved the submitted version.

## Funding

This work was supported by the National Natural Science Foundation of China (81973125, 81941018, 91846303, and 91843302). The CKB baseline survey and the first re-survey were supported by a grant from the Kadoorie Charitable Foundation in Hong Kong. The long-term follow-up was supported by Grants (2016YFC0900500, 2016YFC0900501, 2016YFC0900504, and 2016YFC1303904) from the National Key R&D Program of China, National Natural Science Foundation of China (81390540, 81390541, and 81390544), and Chinese Ministry of Science and Technology (2011BAI09B01). The funders had no role in the study design, data collection, data analysis and interpretation, writing of the report, or the decision to submit the article for publication.

## Conflict of Interest

The authors declare that the research was conducted in the absence of any commercial or financial relationships that could be construed as a potential conflict of interest.

## Publisher's Note

All claims expressed in this article are solely those of the authors and do not necessarily represent those of their affiliated organizations, or those of the publisher, the editors and the reviewers. Any product that may be evaluated in this article, or claim that may be made by its manufacturer, is not guaranteed or endorsed by the publisher.
