# Supplementary material for: Association of Red Meat Consumption, Metabolic Markers, and Risk of Cardiovascular Diseases
Source: Front Nutr. 2022 Apr 15;9:833271. doi: 10.3389/fnut.2022.833271 (PMC9051033; doi:10.3389/fnut.2022.833271)
Supplement: Supplementary file 1 [file Table_1.PDF]

Table S1. Mean and SD values of all 225 metabolites.

Metabolite	Unit	Mean	SD
Mean diameter for VLDL particles	nm	3.75E+01	1.24E+00
Mean diameter for LDL particles	nm	2.35E+01	1.16E-01
Mean diameter for HDL particles	nm	9.99E+00	1.84E-01
Concentration of chylomicrons and extremely large VLDL particles	mol/l	1.43E-10	1.18E-10
Concentration of very large VLDL particles	mol/l	7.94E-10	6.26E-10
Concentration of large VLDL particles	mol/l	5.19E-09	3.33E-09
Concentration of medium VLDL particles	mol/l	1.79E-08	8.18E-09
Concentration of small VLDL particles	mol/l	2.78E-08	8.24E-09
Concentration of very small VLDL particles	mol/l	3.16E-08	6.49E-09
Concentration of IDL particles	mol/l	7.81E-08	1.76E-08
Concentration of large LDL particles	mol/l	1.27E-07	3.15E-08
Concentration of medium LDL particles	mol/l	1.01E-07	2.78E-08
Concentration of small LDL particles	mol/l	1.23E-07	3.08E-08
Concentration of very large HDL particles	mol/l	4.47E-07	1.30E-07
Concentration of large HDL particles	mol/l	1.07E-06	3.36E-07
Concentration of medium HDL particles	mol/l	1.80E-06	2.80E-07
Concentration of small HDL particles	mol/l	4.48E-06	3.82E-07
Total lipids in chylomicrons and extremely large VLDL	mmol/l	3.04E-02	2.51E-02
Total lipids in very large VLDL	mmol/l	7.69E-02	6.11E-02
Total lipids in large VLDL	mmol/l	2.97E-01	1.93E-01
Total lipids in medium VLDL	mmol/l	5.92E-01	2.71E-01
Total lipids in small VLDL	mmol/l	5.40E-01	1.56E-01
Total lipids in very small VLDL	mmol/l	3.98E-01	8.28E-02
Total lipids in IDL	mmol/l	7.86E-01	1.83E-01
Total lipids in large LDL	mmol/l	9.03E-01	2.28E-01
Total lipids in medium LDL	mmol/l	5.15E-01	1.41E-01
Total lipids in small LDL	mmol/l	3.43E-01	8.69E-02
Total lipids in very large HDL	mmol/l	4.53E-01	1.32E-01
Total lipids in large HDL	mmol/l	6.76E-01	2.15E-01
Total lipids in medium HDL	mmol/l	7.60E-01	1.21E-01
Total lipids in small HDL	mmol/l	9.91E-01	8.48E-02
Total cholesterol	mmol/l	3.52E+00	6.12E-01
Remnant cholesterol (non-HDL, non-LDL -cholesterol)	mmol/l	1.09E+00	2.72E-01
Total cholesterol in VLDL	mmol/l	6.07E-01	1.88E-01
Total cholesterol in chylomicrons and extremely large VLDL	mmol/l	5.00E-03	4.14E-03
Total cholesterol in very large VLDL	mmol/l	1.53E-02	1.22E-02
Total cholesterol in large VLDL	mmol/l	6.42E-02	4.39E-02
Total cholesterol in medium VLDL	mmol/l	1.46E-01	6.76E-02
Total cholesterol in small VLDL	mmol/l	1.82E-01	5.14E-02
Total cholesterol in very small VLDL	mmol/l	1.95E-01	4.31E-02
Total cholesterol in IDL	mmol/l	4.84E-01	1.25E-01
Total cholesterol in LDL	mmol/l	1.12E+00	3.49E-01
Total cholesterol in large LDL	mmol/l	5.90E-01	1.72E-01
Total cholesterol in medium LDL	mmol/l	3.30E-01	1.10E-01

Total cholesterol in small LDL	mmol/l	2.05E-01	6.73E-02
Total cholesterol in HDL	mmol/l	1.30E+00	2.12E-01
Total cholesterol in HDL2	mmol/l	8.26E-01	1.97E-01
Total cholesterol in HDL3	mmol/l	4.76E-01	2.02E-02
Total cholesterol in very large HDL	mmol/l	2.21E-01	6.00E-02
Total cholesterol in large HDL	mmol/l	3.23E-01	1.14E-01
Total cholesterol in medium HDL	mmol/l	3.70E-01	6.68E-02
Total cholesterol in small HDL	mmol/l	3.86E-01	5.31E-02
Esterified cholesterol	mmol/l	2.03E+00	4.51E-01
Cholesterol esters in chylomicrons and extremely large VLDL	mmol/l	2.89E-03	2.25E-03
Cholesterol esters in very large VLDL	mmol/l	9.06E-03	6.52E-03
Cholesterol esters in large VLDL	mmol/l	3.60E-02	2.18E-02
Cholesterol esters in medium VLDL	mmol/l	8.06E-02	3.53E-02
Cholesterol esters in small VLDL	mmol/l	1.09E-01	3.38E-02
Cholesterol esters in very small VLDL	mmol/l	1.34E-01	2.97E-02
Cholesterol esters in IDL	mmol/l	3.47E-01	8.92E-02
Cholesterol esters in large LDL	mmol/l	4.15E-01	1.30E-01
Cholesterol esters in medium LDL	mmol/l	2.25E-01	9.13E-02
Cholesterol esters in small LDL	mmol/l	1.42E-01	5.60E-02
Cholesterol esters in very large HDL	mmol/l	1.65E-01	4.25E-02
Cholesterol esters in large HDL	mmol/l	2.53E-01	8.72E-02
Cholesterol esters in medium HDL	mmol/l	3.06E-01	5.33E-02
Cholesterol esters in small HDL	mmol/l	2.85E-01	5.26E-02
Free cholesterol	mmol/l	9.58E-01	1.92E-01
Free cholesterol in chylomicrons and extremely large VLDL	mmol/l	2.12E-03	1.95E-03
Free cholesterol in very large VLDL	mmol/l	6.20E-03	5.73E-03
Free cholesterol in large VLDL	mmol/l	2.81E-02	2.25E-02
Free cholesterol in medium VLDL	mmol/l	6.55E-02	3.39E-02
Free cholesterol in small VLDL	mmol/l	7.28E-02	2.07E-02
Free cholesterol in very small VLDL	mmol/l	6.04E-02	1.44E-02
Free cholesterol in IDL	mmol/l	1.37E-01	3.85E-02
Free cholesterol in large LDL	mmol/l	1.74E-01	4.29E-02
Free cholesterol in medium LDL	mmol/l	1.05E-01	1.99E-02
Free cholesterol in small LDL	mmol/l	6.31E-02	1.23E-02
Free cholesterol in very large HDL	mmol/l	5.68E-02	1.79E-02
Free cholesterol in large HDL	mmol/l	7.02E-02	2.72E-02
Free cholesterol in medium HDL	mmol/l	6.39E-02	1.40E-02
Free cholesterol in small HDL	mmol/l	1.01E-01	1.07E-02
Total triglycerides	mmol/l	1.25E+00	4.77E-01
Triglycerides in VLDL	mmol/l	9.11E-01	4.21E-01
Triglycerides in chylomicrons and extremely large VLDL	mmol/l	2.19E-02	1.78E-02
Triglycerides in very large VLDL	mmol/l	4.99E-02	3.86E-02
Triglycerides in large VLDL	mmol/l	1.82E-01	1.15E-01
Triglycerides in medium VLDL	mmol/l	3.30E-01	1.54E-01
Triglycerides in small VLDL	mmol/l	2.32E-01	8.17E-02
Triglycerides in very small VLDL	mmol/l	9.35E-02	2.43E-02

Triglycerides in IDL	mmol/l	8.58E-02	2.19E-02
Triglycerides in LDL	mmol/l	1.32E-01	3.47E-02
Triglycerides in large LDL	mmol/l	7.15E-02	1.93E-02
Triglycerides in medium LDL	mmol/l	3.53E-02	9.71E-03
Triglycerides in small LDL	mmol/l	2.48E-02	7.03E-03
Triglycerides in HDL	mmol/l	1.21E-01	3.17E-02
Triglycerides in very large HDL	mmol/l	1.54E-02	6.53E-03
Triglycerides in large HDL	mmol/l	2.23E-02	1.07E-02
Triglycerides in medium HDL	mmol/l	3.88E-02	1.03E-02
Triglycerides in small HDL	mmol/l	4.46E-02	1.13E-02
Sphingomyelins	mmol/l	3.01E-01	5.71E-02
Total phosphoglycerides	mmol/l	1.70E+00	2.36E-01
Total cholines	mmol/l	1.94E+00	2.51E-01
Phosphatidylcholine and other cholines	mmol/l	1.50E+00	2.26E-01
Phospholipids in chylomicrons and extremely large VLDL	mmol/l	3.57E-03	3.24E-03
Phospholipids in very large VLDL	mmol/l	1.17E-02	1.04E-02
Phospholipids in large VLDL	mmol/l	5.16E-02	3.50E-02
Phospholipids in medium VLDL	mmol/l	1.16E-01	5.19E-02
Phospholipids in small VLDL	mmol/l	1.25E-01	3.31E-02
Phospholipids in very small VLDL	mmol/l	1.10E-01	2.73E-02
Phospholipids in IDL	mmol/l	2.16E-01	4.77E-02
Phospholipids in large LDL	mmol/l	2.41E-01	4.75E-02
Phospholipids in medium LDL	mmol/l	1.50E-01	2.83E-02
Phospholipids in small LDL	mmol/l	1.13E-01	1.90E-02
Phospholipids in very large HDL	mmol/l	2.16E-01	7.41E-02
Phospholipids in large HDL	mmol/l	3.31E-01	9.48E-02
Phospholipids in medium HDL	mmol/l	3.51E-01	5.39E-02
Phospholipids in small HDL	mmol/l	5.60E-01	6.35E-02
Apolipoprotein A-I	g/l	1.41E+00	1.22E-01
Apolipoprotein B	g/l	7.37E-01	1.37E-01
Ratio of apolipoprotein B to apolipoprotein A-I		5.26E-01	9.87E-02
Total fatty acids	mmol/l	9.82E+00	1.95E+00
Saturated fatty acids	mmol/l	3.69E+00	7.02E-01
Monounsaturated fatty acids; 16:1, 18:1	mmol/l	2.90E+00	6.81E-01
Polyunsaturated fatty acids	mmol/l	3.23E+00	6.55E-01
Omega-3 fatty acids	mmol/l	4.32E-01	1.21E-01
22:6, docosahexaenoic acid	mmol/l	1.54E-01	3.37E-02
Omega-6 fatty acids	mmol/l	2.80E+00	5.58E-01
18:2, linoleic acid	mmol/l	2.40E+00	5.52E-01
Ratio of saturated fatty acids to total fatty acids	%	3.77E+01	1.69E+00
Ratio of monounsaturated fatty acids to total fatty acids	%	2.94E+01	1.97E+00
Ratio of polyunsaturated fatty acids to total fatty acids	%	3.29E+01	2.57E+00
Ratio of omega-3 fatty acids to total fatty acids	%	4.38E+00	6.57E-01
Ratio of 22:6 docosahexaenoic acid to total fatty acids	%	1.58E+00	2.39E-01
Ratio of omega-6 fatty acids to total fatty acids	%	2.85E+01	2.52E+00
Ratio of 18:2 linoleic acid to total fatty acids	%	2.44E+01	3.43E+00

Alanine	mmol/l	4.01E-01	6.12E-02
Glutamine	mmol/l	4.66E-01	6.46E-02
Histidine	mmol/l	6.27E-02	6.37E-03
Isoleucine	mmol/l	6.06E-02	1.91E-02
Leucine	mmol/l	7.65E-02	2.03E-02
Phenylalanine	mmol/l	6.53E-02	1.01E-02
Tyrosine	mmol/l	5.41E-02	1.14E-02
Valine	mmol/l	1.68E-01	3.84E-02
Citrate	mmol/l	1.28E-01	2.20E-02
Glucose	mmol/l	4.22E+00	2.29E+00
Lactate	mmol/l	2.56E+00	7.93E-01
Glycoprotein acetyls, mainly a1-acid glycoprotein	mmol/l	1.35E+00	2.27E-01
Acetoacetate	mmol/l	2.45E-02	1.51E-02
Acetate	mmol/l	5.37E-02	8.42E-02
3-hydroxybutyrate	mmol/l	1.10E-01	4.86E-02
Cholesterol esters to total lipids ratio in IDL	%	4.39E+01	2.19E+00
Cholesterol esters to total lipids ratio in chylomicrons and extremely large VLI	%	9.41E+00	4.40E+00
Cholesterol esters to total lipids ratio in large HDL	%	3.71E+01	1.73E+00
Cholesterol esters to total lipids ratio in large LDL	%	4.52E+01	4.04E+00
Cholesterol esters to total lipids ratio in large VLDL	%	1.22E+01	2.39E+00
Cholesterol esters to total lipids ratio in medium HDL	%	4.02E+01	2.18E+00
Cholesterol esters to total lipids ratio in medium LDL	%	4.18E+01	8.35E+00
Cholesterol esters to total lipids ratio in medium VLDL	%	1.38E+01	2.43E+00
Cholesterol esters to total lipids ratio in small HDL	%	2.88E+01	4.79E+00
Cholesterol esters to total lipids ratio in small LDL	%	3.98E+01	8.36E+00
Cholesterol esters to total lipids ratio in small VLDL	%	2.05E+01	4.15E+00
Cholesterol esters to total lipids ratio in very large HDL	%	3.69E+01	3.82E+00
Cholesterol esters to total lipids ratio in very large VLDL	%	1.19E+01	3.76E+00
Cholesterol esters to total lipids ratio in very small VLDL	%	3.37E+01	2.87E+00
Free cholesterol to total lipids ratio in IDL	%	1.73E+01	2.06E+00
Free cholesterol to total lipids ratio in chylomicrons and extremely large VLDI	%	6.09E+00	2.14E+00
Free cholesterol to total lipids ratio in large HDL	%	1.02E+01	1.05E+00
Free cholesterol to total lipids ratio in large LDL	%	1.94E+01	1.33E+00
Free cholesterol to total lipids ratio in large VLDL	%	8.40E+00	2.24E+00
Free cholesterol to total lipids ratio in medium HDL	%	8.33E+00	6.13E-01
Free cholesterol to total lipids ratio in medium LDL	%	2.08E+01	2.16E+00
Free cholesterol to total lipids ratio in medium VLDL	%	1.08E+01	8.78E-01
Free cholesterol to total lipids ratio in small HDL	%	1.02E+01	5.46E-01
Free cholesterol to total lipids ratio in small LDL	%	1.86E+01	1.55E+00
Free cholesterol to total lipids ratio in small VLDL	%	1.35E+01	5.78E-01
Free cholesterol to total lipids ratio in very large HDL	%	1.25E+01	7.59E-01
Free cholesterol to total lipids ratio in very large VLDL	%	6.79E+00	2.53E+00
Free cholesterol to total lipids ratio in very small VLDL	%	1.51E+01	1.42E+00
Phospholipids to total lipds ratio in IDL	%	2.76E+01	9.87E-01
Phospholipids to total lipds ratio in chylomicrons and extremely large VLDL	%	1.04E+01	3.05E+00
Phospholipids to total lipds ratio in large HDL	%	4.94E+01	2.48E+00

Phospholipids to total lipds ratio in large LDL	%	2.71E+01	1.94E+00
Phospholipids to total lipds ratio in large VLDL	%	1.69E+01	1.12E+00
Phospholipids to total lipds ratio in medium HDL	%	4.63E+01	1.44E+00
Phospholipids to total lipds ratio in medium LDL	%	2.98E+01	4.00E+00
Phospholipids to total lipds ratio in medium VLDL	%	1.96E+01	3.59E-01
Phospholipids to total lipds ratio in small HDL	%	5.65E+01	3.69E+00
Phospholipids to total lipds ratio in small LDL	%	3.36E+01	4.49E+00
Phospholipids to total lipds ratio in small VLDL	%	2.34E+01	1.02E+00
Phospholipids to total lipds ratio in very large HDL	%	4.71E+01	5.11E+00
Phospholipids to total lipds ratio in very large VLDL	%	1.32E+01	4.17E+00
Phospholipids to total lipds ratio in very small VLDL	%	2.74E+01	2.62E+00
Total cholesterol to total lipids ratio in IDL	%	6.12E+01	2.77E+00
Total cholesterol to total lipids ratio in chylomicrons and extremely large VLD	%	1.55E+01	5.58E+00
Total cholesterol to total lipids ratio in large HDL	%	4.72E+01	2.52E+00
Total cholesterol to total lipids ratio in large LDL	%	6.46E+01	4.18E+00
Total cholesterol to total lipids ratio in large VLDL	%	2.06E+01	3.43E+00
Total cholesterol to total lipids ratio in medium HDL	%	4.85E+01	2.49E+00
Total cholesterol to total lipids ratio in medium LDL	%	6.27E+01	6.40E+00
Total cholesterol to total lipids ratio in medium VLDL	%	2.46E+01	2.47E+00
Total cholesterol to total lipids ratio in small HDL	%	3.90E+01	4.33E+00
Total cholesterol to total lipids ratio in small LDL	%	5.85E+01	7.08E+00
Total cholesterol to total lipids ratio in small VLDL	%	3.41E+01	4.39E+00
Total cholesterol to total lipids ratio in very large HDL	%	4.93E+01	4.17E+00
Total cholesterol to total lipids ratio in very large VLDL	%	1.87E+01	5.17E+00
Total cholesterol to total lipids ratio in very small VLDL	%	4.88E+01	3.40E+00
Triglycerides to total lipids ratio in IDL	%	1.12E+01	2.85E+00
Triglycerides to total lipids ratio in chylomicrons and extremely large VLDL	%	6.97E+01	1.55E+01
Triglycerides to total lipids ratio in large HDL	%	3.33E+00	1.31E+00
Triglycerides to total lipids ratio in large LDL	%	8.19E+00	2.36E+00
Triglycerides to total lipids ratio in large VLDL	%	6.19E+01	3.50E+00
Triglycerides to total lipids ratio in medium HDL	%	5.18E+00	1.46E+00
Triglycerides to total lipids ratio in medium LDL	%	7.09E+00	2.10E+00
Triglycerides to total lipids ratio in medium VLDL	%	5.58E+01	2.67E+00
Triglycerides to total lipids ratio in small HDL	%	4.51E+00	1.16E+00
Triglycerides to total lipids ratio in small LDL	%	7.44E+00	2.26E+00
Triglycerides to total lipids ratio in small VLDL	%	4.25E+01	4.58E+00
Triglycerides to total lipids ratio in very large HDL	%	3.51E+00	1.46E+00
Triglycerides to total lipids ratio in very large VLDL	%	6.49E+01	5.97E+00
Triglycerides to total lipids ratio in very small VLDL	%	2.37E+01	4.97E+00
Ratio of triglycerides to phosphoglycerides		7.16E-01	2.42E-01
Albumin	signal area	8.51E-02	2.95E-03
Creatinine	mmol/l	5.33E-02	2.55E-02
Estimated degree of unsaturation		1.07E+00	6.16E-02
