# Supplementary material for: Association of Red Meat Consumption, Metabolic Markers, and Risk of Cardiovascular Diseases
Source: Front Nutr. 2022 Apr 15;9:833271. doi: 10.3389/fnut.2022.833271 (PMC9051033; doi:10.3389/fnut.2022.833271)
Supplement: Supplementary file 2 [file Table_2.PDF]

Table S2. Associations (95% CI) of red meat consumption with all 225 log-transformed metabolic markers or devided traits, and of these markers with ris

Metabolic marker	n	Coefficient (95% CI)	*p*	FDR-*p*	OR (95% CI)	*p*	FDR-*p*
Mean diameter for VLDL particles	4,777	0.006 (-0.008, 0.020)	0.412	0.555	1.086 (1.010, 1.167)	0.026	0.054
Mean diameter for LDL particles	4,777	-0.013 (-0.026, 0.001)	0.067	0.176	0.885 (0.818, 0.956)	0.002	0.008
Mean diameter for HDL particles	4,777	0.003 (-0.011, 0.017)	0.654	0.729	0.897 (0.832, 0.967)	0.004	0.015
Concentration of chylomicrons and extremely large VLDL particles	4,778	0.014 (0.000, 0.029)	0.056	0.168	1.069 (0.997, 1.147)	0.061	0.108
Concentration of very large VLDL particles	4,778	0.011 (-0.003, 0.026)	0.121	0.236	1.090 (1.015, 1.170)	0.018	0.042
Concentration of large VLDL particles	4,778	0.010 (-0.004, 0.025)	0.162	0.278	1.118 (1.040, 1.201)	0.002	0.009
Concentration of medium VLDL particles	4,778	0.012 (-0.002, 0.026)	0.104	0.223	1.135 (1.056, 1.221)	0.001	0.004
Concentration of small VLDL particles	4,778	0.011 (-0.003, 0.025)	0.139	0.255	1.155 (1.074, 1.243)	<0.001	0.003
Concentration of very small VLDL particles	4,778	0.017 (0.003, 0.031)	0.015	0.068	1.143 (1.061, 1.232)	<0.001	0.004
Concentration of IDL particles	4,777	0.023 (0.009, 0.037)	0.001	0.010	1.102 (1.022, 1.188)	0.011	0.030
Concentration of large LDL particles	4,778	0.022 (0.008, 0.036)	0.002	0.012	1.114 (1.034, 1.201)	0.005	0.015
Concentration of medium LDL particles	4,778	0.023 (0.009, 0.037)	0.001	0.012	1.129 (1.048, 1.216)	0.001	0.006
Concentration of small LDL particles	4,778	0.025 (0.011, 0.039)	<0.001	0.006	1.142 (1.059, 1.230)	0.001	0.004
Concentration of very large HDL particles	4,778	0.016 (0.002, 0.031)	0.022	0.085	0.961 (0.893, 1.035)	0.295	0.390
Concentration of large HDL particles	4,778	0.005 (-0.009, 0.018)	0.490	0.616	0.883 (0.818, 0.954)	0.002	0.007
Concentration of medium HDL particles	4,778	0.018 (0.004, 0.031)	0.011	0.054	0.924 (0.856, 0.998)	0.044	0.085
Concentration of small HDL particles	4,778	0.019 (0.005, 0.033)	0.007	0.042	1.024 (0.949, 1.105)	0.539	0.629
Total lipids in chylomicrons and extremely large VLDL	4,778	0.014 (0.000, 0.029)	0.056	0.167	1.070 (0.998, 1.148)	0.057	0.105
Total lipids in very large VLDL	4,778	0.011 (-0.003, 0.026)	0.123	0.239	1.090 (1.015, 1.170)	0.018	0.042
Total lipids in large VLDL	4,778	0.010 (-0.004, 0.024)	0.166	0.279	1.116 (1.039, 1.199)	0.003	0.009
Total lipids in medium VLDL	4,778	0.012 (-0.002, 0.026)	0.103	0.225	1.135 (1.056, 1.220)	0.001	0.004
Total lipids in small VLDL	4,778	0.011 (-0.003, 0.025)	0.129	0.244	1.157 (1.075, 1.244)	<0.001	0.003
Total lipids in very small VLDL	4,778	0.017 (0.003, 0.031)	0.017	0.073	1.135 (1.053, 1.223)	0.001	0.005
Total lipids in IDL	4,777	0.023 (0.009, 0.037)	0.001	0.012	1.096 (1.017, 1.182)	0.017	0.039
Total lipids in large LDL	4,778	0.022 (0.008, 0.036)	0.002	0.015	1.109 (1.029, 1.196)	0.007	0.020
Total lipids in medium LDL	4,778	0.023 (0.009, 0.036)	0.001	0.012	1.126 (1.045, 1.213)	0.002	0.007
Total lipids in small LDL	4,778	0.025 (0.011, 0.038)	0.001	0.006	1.136 (1.054, 1.224)	0.001	0.004
Total lipids in very large HDL	4,778	0.017 (0.003, 0.031)	0.018	0.078	0.962 (0.894, 1.036)	0.305	0.399
Total lipids in large HDL	4,778	0.004 (-0.009, 0.018)	0.516	0.638	0.882 (0.817, 0.953)	0.001	0.006
Total lipids in medium HDL	4,778	0.017 (0.004, 0.031)	0.014	0.063	0.922 (0.853, 0.995)	0.038	0.074
Total lipids in small HDL	4,778	0.019 (0.005, 0.032)	0.008	0.044	1.020 (0.945, 1.100)	0.611	0.702
Total cholesterol	4,777	0.028 (0.015, 0.042)	<0.001	0.002	1.108 (1.028, 1.195)	0.007	0.021
Total cholesterol in VLDL	4,777	0.023 (0.009, 0.037)	0.001	0.010	1.152 (1.069, 1.242)	<0.001	0.002
Total cholesterol in chylomicrons and extremely large VLDL	4,777	0.016 (0.002, 0.031)	0.022	0.087	1.151 (1.070, 1.239)	<0.001	0.003
Total cholesterol in very large VLDL	4,778	0.013 (-0.001, 0.028)	0.076	0.183	1.072 (0.999, 1.150)	0.053	0.099
Total cholesterol in large VLDL	4,778	0.010 (-0.004, 0.025)	0.158	0.273	1.071 (0.998, 1.149)	0.058	0.105
Total cholesterol in medium VLDL	4,778	0.008 (-0.006, 0.022)	0.268	0.413	1.095 (1.020, 1.175)	0.012	0.032
Total cholesterol in small VLDL	4,778	0.013 (-0.001, 0.027)	0.074	0.182	1.122 (1.044, 1.206)	0.002	0.007
Total cholesterol in very small VLDL	4,778	0.012 (-0.002, 0.026)	0.105	0.222	1.151 (1.069, 1.239)	<0.001	0.003
Total cholesterol in IDL	4,778	0.015 (0.001, 0.029)	0.040	0.129	1.096 (1.017, 1.182)	0.017	0.040
Total cholesterol in LDL	4,778	0.023 (0.009, 0.037)	0.001	0.011	1.084 (1.006, 1.169)	0.035	0.069
Total cholesterol in large LDL	4,777	0.018 (0.004, 0.032)	0.012	0.056	1.110 (1.028, 1.198)	0.008	0.022
Total cholesterol in medium LDL	4,778	0.019 (0.005, 0.033)	0.008	0.044	1.102 (1.022, 1.189)	0.011	0.030
Total cholesterol in small LDL	4,778	0.019 (0.005, 0.033)	0.008	0.043	1.100 (1.022, 1.184)	0.012	0.031
Total cholesterol in HDL	4,778	0.019 (0.005, 0.033)	0.008	0.042	1.099 (1.021, 1.183)	0.012	0.032
Total cholesterol in HDL2	4,777	0.015 (0.001, 0.029)	0.032	0.116	0.912 (0.846, 0.983)	0.016	0.038
Total cholesterol in HDL3	4,777	0.013 (-0.001, 0.027)	0.070	0.180	0.897 (0.833, 0.967)	0.005	0.015
Total cholesterol in very large HDL	4,777	0.021 (0.007, 0.034)	0.004	0.026	1.061 (0.984, 1.144)	0.121	0.184
Total cholesterol in large HDL	4,778	0.022 (0.008, 0.037)	0.002	0.014	1.001 (0.931, 1.077)	0.978	0.978
Total cholesterol in medium HDL	4,778	0.003 (-0.010, 0.017)	0.625	0.714	0.877 (0.812, 0.947)	0.001	0.004
Total cholesterol in small HDL	4,778	0.013 (-0.001, 0.027)	0.059	0.171	0.915 (0.847, 0.988)	0.024	0.051
Remnant cholesterol (non-HDL, non-LDL -cholesterol)	4,778	0.008 (-0.006, 0.022)	0.280	0.422	1.032 (0.959, 1.111)	0.398	0.506
Esterified cholesterol	4,774	0.005 (-0.010, 0.020)	0.512	0.636	0.985 (0.899, 1.079)	0.745	0.810
Cholesterol esters in chylomicrons and extremely large VLDL	4,778	0.013 (-0.002, 0.027)	0.079	0.187	1.071 (0.999, 1.150)	0.055	0.101
Cholesterol esters in very large VLDL	4,778	0.010 (-0.005, 0.024)	0.180	0.295	1.062 (0.989, 1.139)	0.096	0.153
Cholesterol esters in large VLDL	4,778	0.006 (-0.008, 0.020)	0.417	0.556	1.064 (0.993, 1.140)	0.077	0.131
Cholesterol esters in medium VLDL	4,778	0.013 (-0.001, 0.028)	0.063	0.172	1.105 (1.028, 1.188)	0.007	0.020
Cholesterol esters in small VLDL	4,778	0.010 (-0.004, 0.024)	0.151	0.264	1.130 (1.049, 1.217)	0.001	0.006
Cholesterol esters in very small VLDL	4,778	0.015 (0.001, 0.029)	0.033	0.115	1.090 (1.011, 1.176)	0.025	0.053
Cholesterol esters in IDL	4,778	0.025 (0.012, 0.039)	<0.001	0.004	1.097 (1.018, 1.183)	0.016	0.039
Cholesterol esters in large LDL	4,778	0.014 (0.000, 0.028)	0.050	0.153	1.121 (1.035, 1.214)	0.005	0.017
Cholesterol esters in medium LDL	4,778	0.014 (-0.001, 0.028)	0.060	0.167	1.073 (0.997, 1.155)	0.060	0.106
Cholesterol esters in small LDL	4,778	0.011 (-0.003, 0.025)	0.125	0.240	1.065 (0.990, 1.146)	0.091	0.148
Cholesterol esters in very large HDL	4,778	0.023 (0.009, 0.037)	0.002	0.012	1.019 (0.947, 1.095)	0.620	0.708
Cholesterol esters in large HDL	4,778	0.004 (-0.010, 0.017)	0.581	0.695	0.875 (0.811, 0.945)	0.001	0.004
Cholesterol esters in medium HDL	4,778	0.013 (-0.001, 0.027)	0.069	0.178	0.916 (0.848, 0.990)	0.026	0.054
Cholesterol esters in small HDL	4,778	0.002 (-0.012, 0.016)	0.802	0.867	1.026 (0.953, 1.105)	0.493	0.593

Free cholesterol	4,774	0.029 (0.016, 0.043)	<0.001	0.001	1.105 (1.026, 1.191)	0.008	0.024
Free cholesterol in chylomicrons and extremely large VLDL	4,778	0.011 (-0.004, 0.025)	0.143	0.255	1.069 (0.997, 1.147)	0.062	0.109
Free cholesterol in very large VLDL	4,778	0.011 (-0.003, 0.025)	0.134	0.249	1.064 (0.992, 1.142)	0.082	0.137
Free cholesterol in large VLDL	4,778	0.008 (-0.006, 0.022)	0.278	0.423	1.099 (1.024, 1.179)	0.009	0.024
Free cholesterol in medium VLDL	4,778	0.011 (-0.003, 0.026)	0.118	0.236	1.131 (1.053, 1.216)	0.001	0.004
Free cholesterol in small VLDL	4,778	0.011 (-0.003, 0.025)	0.131	0.246	1.154 (1.073, 1.242)	<0.001	0.003
Free cholesterol in very small VLDL	4,778	0.011 (-0.003, 0.026)	0.114	0.238	1.099 (1.021, 1.184)	0.012	0.032
Free cholesterol in IDL	4,778	0.012 (-0.002, 0.027)	0.088	0.197	1.050 (0.974, 1.131)	0.204	0.279
Free cholesterol in large LDL	4,778	0.016 (0.002, 0.030)	0.025	0.092	1.070 (0.993, 1.153)	0.077	0.132
Free cholesterol in medium LDL	4,778	0.026 (0.012, 0.040)	<0.001	0.003	1.119 (1.038, 1.206)	0.003	0.011
Free cholesterol in small LDL	4,778	0.028 (0.014, 0.042)	<0.001	0.002	1.124 (1.043, 1.211)	0.002	0.008
Free cholesterol in very large HDL	4,778	0.020 (0.006, 0.034)	0.005	0.032	0.960 (0.892, 1.033)	0.276	0.367
Free cholesterol in large HDL	4,778	0.001 (-0.013, 0.015)	0.864	0.917	0.890 (0.824, 0.962)	0.003	0.011
Free cholesterol in medium HDL	4,778	0.015 (0.001, 0.028)	0.035	0.120	0.914 (0.847, 0.987)	0.022	0.047
Free cholesterol in small HDL	4,778	0.016 (0.002, 0.030)	0.021	0.085	0.972 (0.901, 1.050)	0.472	0.577
Total triglycerides	4,777	0.012 (-0.002, 0.027)	0.086	0.196	1.141 (1.061, 1.227)	<0.001	0.003
Triglycerides in VLDL	4,777	0.011 (-0.003, 0.026)	0.119	0.235	1.135 (1.056, 1.221)	0.001	0.004
Triglycerides in chylomicrons and extremely large VLDL	4,778	0.014 (-0.001, 0.028)	0.065	0.174	1.061 (0.990, 1.138)	0.094	0.151
Triglycerides in very large VLDL	4,778	0.012 (-0.003, 0.026)	0.116	0.240	1.089 (1.014, 1.170)	0.019	0.041
Triglycerides in large VLDL	4,778	0.011 (-0.004, 0.025)	0.148	0.260	1.121 (1.044, 1.205)	0.002	0.007
Triglycerides in medium VLDL	4,778	0.011 (-0.003, 0.025)	0.127	0.242	1.138 (1.058, 1.223)	<0.001	0.004
Triglycerides in small VLDL	4,778	0.008 (-0.006, 0.023)	0.257	0.398	1.146 (1.066, 1.233)	<0.001	0.002
Triglycerides in very small VLDL	4,778	0.008 (-0.006, 0.022)	0.257	0.401	1.147 (1.066, 1.235)	<0.001	0.002
Triglycerides in IDL	4,778	0.010 (-0.004, 0.024)	0.148	0.261	1.141 (1.058, 1.230)	0.001	0.004
Triglycerides in LDL	4,777	0.016 (0.002, 0.030)	0.028	0.100	1.141 (1.058, 1.230)	0.001	0.004
Triglycerides in large LDL	4,778	0.013 (-0.001, 0.026)	0.078	0.187	1.135 (1.051, 1.225)	0.001	0.005
Triglycerides in medium LDL	4,778	0.015 (0.001, 0.029)	0.041	0.128	1.152 (1.069, 1.241)	<0.001	0.003
Triglycerides in small LDL	4,778	0.017 (0.002, 0.031)	0.023	0.087	1.179 (1.095, 1.269)	<0.001	0.001
Triglycerides in HDL	4,777	0.010 (-0.003, 0.024)	0.142	0.256	1.057 (0.981, 1.139)	0.145	0.209
Triglycerides in very large HDL	4,778	0.012 (-0.002, 0.026)	0.094	0.209	1.068 (0.991, 1.151)	0.086	0.142
Triglycerides in large HDL	4,778	0.004 (-0.010, 0.017)	0.599	0.713	0.974 (0.899, 1.055)	0.514	0.609
Triglycerides in medium HDL	4,778	0.012 (-0.002, 0.026)	0.103	0.223	1.045 (0.971, 1.123)	0.238	0.321
Triglycerides in small HDL	4,778	0.006 (-0.008, 0.021)	0.393	0.543	1.101 (1.024, 1.184)	0.010	0.026
Phospholipids in chylomicrons and extremely large VLDL	4,771	0.034 (0.020, 0.048)	<0.001	<0.001	1.052 (0.977, 1.132)	0.177	0.243
Phospholipids in very large VLDL	4,771	0.031 (0.016, 0.045)	<0.001	0.001	1.094 (1.017, 1.178)	0.017	0.039
Phospholipids in large VLDL	4,771	0.031 (0.016, 0.045)	<0.001	0.001	1.089 (1.011, 1.172)	0.024	0.051
Phospholipids in medium VLDL	4,771	0.028 (0.014, 0.043)	<0.001	0.002	1.062 (0.987, 1.142)	0.106	0.165
Phospholipids in small VLDL	4,778	0.013 (-0.001, 0.028)	0.073	0.182	1.067 (0.995, 1.145)	0.069	0.120
Phospholipids in very small VLDL	4,778	0.012 (-0.003, 0.026)	0.117	0.238	1.066 (0.994, 1.144)	0.073	0.126
Phospholipids in IDL	4,778	0.010 (-0.004, 0.024)	0.174	0.288	1.116 (1.039, 1.198)	0.003	0.009
Phospholipids in large LDL	4,778	0.012 (-0.002, 0.026)	0.095	0.209	1.136 (1.057, 1.221)	0.001	0.004
Phospholipids in medium LDL	4,778	0.011 (-0.003, 0.025)	0.118	0.236	1.142 (1.062, 1.229)	<0.001	0.003
Phospholipids in small LDL	4,778	0.017 (0.003, 0.031)	0.021	0.085	1.108 (1.029, 1.195)	0.007	0.021
Phospholipids in very large HDL	4,778	0.020 (0.006, 0.034)	0.004	0.025	1.078 (1.001, 1.162)	0.047	0.090
Phospholipids in large HDL	4,778	0.025 (0.011, 0.038)	0.001	0.006	1.113 (1.032, 1.200)	0.005	0.017
Phospholipids in medium HDL	4,778	0.030 (0.017, 0.044)	<0.001	0.001	1.156 (1.072, 1.247)	<0.001	0.003
Phospholipids in small HDL	4,778	0.033 (0.019, 0.047)	<0.001	<0.001	1.155 (1.070, 1.246)	<0.001	0.003
Apolipoprotein A-I	4,778	0.009 (-0.005, 0.023)	0.220	0.351	0.927 (0.861, 0.999)	0.046	0.088
Apolipoprotein B	4,778	0.005 (-0.008, 0.019)	0.459	0.590	0.890 (0.824, 0.961)	0.003	0.010
Ratio of apolipoprotein B to apolipoprotein A-I	4,778	0.018 (0.004, 0.031)	0.009	0.047	0.926 (0.857, 1.000)	0.051	0.096
Total fatty acids	4,778	0.015 (0.001, 0.029)	0.033	0.115	0.977 (0.906, 1.054)	0.550	0.638
Saturated fatty acids	4,777	0.025 (0.012, 0.039)	<0.001	0.004	0.989 (0.917, 1.067)	0.782	0.830
Monounsaturated fatty acids; 16:1, 18:1	4,775	0.025 (0.011, 0.039)	<0.001	0.004	1.186 (1.100, 1.279)	<0.001	0.001
Polyunsaturated fatty acids	4,775	0.013 (-0.001, 0.027)	0.068	0.177	1.188 (1.102, 1.281)	<0.001	0.001
Omega-3 fatty acids	4,769	0.028 (0.014, 0.042)	<0.001	0.002	1.151 (1.069, 1.240)	<0.001	0.003
22:6, docosahexaenoic acid	4,769	0.029 (0.015, 0.043)	<0.001	0.002	1.131 (1.051, 1.217)	0.001	0.005
Omega-6 fatty acids	4,769	0.024 (0.010, 0.038)	0.001	0.009	1.149 (1.067, 1.236)	<0.001	0.003
18:2, linoleic acid	4,769	0.026 (0.013, 0.040)	<0.001	0.003	1.156 (1.071, 1.248)	<0.001	0.003
Ratio of saturated fatty acids to total fatty acids	4,769	0.014 (0.000, 0.027)	0.050	0.153	1.143 (1.058, 1.235)	0.001	0.004
Ratio of monounsaturated fatty acids to total fatty acids	4,769	0.012 (-0.002, 0.025)	0.083	0.191	1.152 (1.067, 1.243)	<0.001	0.003
Ratio of polyunsaturated fatty acids to total fatty acids	4,769	0.028 (0.014, 0.042)	<0.001	0.002	1.152 (1.067, 1.243)	<0.001	0.003
Ratio of omega-3 fatty acids to total fatty acids	4,769	0.019 (0.005, 0.033)	0.010	0.049	0.991 (0.910, 1.079)	0.837	0.868
Ratio of 22:6 docosahexaenoic acid to total fatty acids	4,778	-0.002 (-0.016, 0.012)	0.754	0.824	0.911 (0.843, 0.984)	0.018	0.041
Ratio of omega-6 fatty acids to total fatty acids	4,778	0.000 (-0.014, 0.014)	0.955	0.972	1.062 (0.986, 1.144)	0.114	0.175
Ratio of 18:2 linoleic acid to total fatty acids	4,778	0.001 (-0.012, 0.015)	0.864	0.912	1.010 (0.937, 1.088)	0.798	0.839
Alanine	4,778	-0.013 (-0.026, 0.001)	0.060	0.169	1.033 (0.956, 1.116)	0.416	0.522
Glutamine	4,778	-0.019 (-0.032, -0.005)	0.009	0.046	0.997 (0.929, 1.071)	0.937	0.950
Histidine	4,778	0.004 (-0.010, 0.018)	0.542	0.656	1.005 (0.934, 1.080)	0.903	0.924
Isoleucine	4,778	0.010 (-0.004, 0.025)	0.163	0.276	0.973 (0.903, 1.049)	0.480	0.584

Leucine	4,776	-0.006 (-0.020, 0.009)	0.439	0.574	1.003 (0.933, 1.078)	0.943	0.952
Phenylalanine	4,758	0.007 (-0.007, 0.021)	0.338	0.485	0.918 (0.850, 0.991)	0.029	0.059
Tyrosine	4,768	0.013 (-0.001, 0.027)	0.075	0.182	0.988 (0.920, 1.062)	0.751	0.808
Valine	4,776	0.012 (-0.001, 0.026)	0.080	0.187	1.015 (0.942, 1.093)	0.700	0.780
Citrate	4,777	0.016 (0.002, 0.029)	0.025	0.092	1.013 (0.938, 1.094)	0.749	0.811
Glucose	4,777	-0.005 (-0.019, 0.008)	0.440	0.572	0.995 (0.921, 1.076)	0.910	0.926
Lactate	4,768	-0.004 (-0.018, 0.010)	0.601	0.712	0.994 (0.922, 1.071)	0.872	0.896
Glycoprotein acetyls, mainly a1-acid glycoprotein	4,755	0.013 (-0.001, 0.027)	0.060	0.169	1.002 (0.928, 1.081)	0.968	0.973
Acetoacetate	4,778	-0.005 (-0.019, 0.009)	0.518	0.637	0.943 (0.874, 1.016)	0.122	0.184
Acetate	4,766	0.006 (-0.008, 0.020)	0.388	0.539	1.236 (1.143, 1.336)	<0.001	<0.001
3-hydroxybutyrate	4,777	-0.011 (-0.025, 0.003)	0.111	0.233	1.055 (0.979, 1.138)	0.160	0.227
Cholesterol esters to total lipids ratio in IDL	4,777	0.005 (-0.009, 0.019)	0.479	0.609	1.274 (1.179, 1.377)	<0.001	<0.001
Cholesterol esters to total lipids ratio in chylomicrons and extremely large VLDL	4,777	0.012 (-0.002, 0.026)	0.083	0.193	1.053 (0.978, 1.134)	0.170	0.238
Cholesterol esters to total lipids ratio in large HDL	4,777	0.004 (-0.010, 0.017)	0.603	0.706	0.980 (0.902, 1.064)	0.628	0.714
Cholesterol esters to total lipids ratio in large LDL	4,667	0.014 (0.000, 0.027)	0.049	0.154	1.162 (1.069, 1.263)	<0.001	0.004
Cholesterol esters to total lipids ratio in large VLDL	4,778	0.026 (0.012, 0.040)	<0.001	0.005	1.060 (0.983, 1.143)	0.130	0.192
Cholesterol esters to total lipids ratio in medium HDL	4,778	0.009 (-0.005, 0.024)	0.209	0.335	1.054 (0.982, 1.131)	0.145	0.210
Cholesterol esters to total lipids ratio in medium LDL	4,778	-0.002 (-0.016, 0.012)	0.753	0.826	0.872 (0.808, 0.942)	<0.001	0.004
Cholesterol esters to total lipids ratio in medium VLDL	4,778	0.000 (-0.015, 0.014)	0.993	0.993	1.114 (1.006, 1.233)	0.037	0.074
Cholesterol esters to total lipids ratio in small HDL	4,778	-0.001 (-0.015, 0.014)	0.911	0.940	0.994 (0.928, 1.064)	0.862	0.889
Cholesterol esters to total lipids ratio in small LDL	4,778	-0.006 (-0.020, 0.008)	0.429	0.565	0.941 (0.868, 1.020)	0.142	0.207
Cholesterol esters to total lipids ratio in small VLDL	4,778	0.006 (-0.009, 0.020)	0.452	0.584	1.034 (0.960, 1.113)	0.378	0.484
Cholesterol esters to total lipids ratio in very large HDL	4,778	0.005 (-0.009, 0.019)	0.490	0.619	0.942 (0.872, 1.017)	0.126	0.189
Cholesterol esters to total lipids ratio in very large VLDL	4,778	-0.004 (-0.019, 0.010)	0.564	0.678	1.022 (0.949, 1.101)	0.567	0.654
Cholesterol esters to total lipids ratio in very small VLDL	4,778	0.003 (-0.012, 0.017)	0.731	0.806	1.026 (0.953, 1.105)	0.489	0.591
Free cholesterol to total lipids ratio in IDL	4,778	-0.001 (-0.015, 0.013)	0.866	0.911	0.987 (0.916, 1.064)	0.737	0.805
Free cholesterol to total lipids ratio in chylomicrons and extremely large VLDL	4,778	0.011 (-0.003, 0.024)	0.118	0.238	1.186 (1.098, 1.282)	<0.001	0.001
Free cholesterol to total lipids ratio in large HDL	4,778	0.005 (-0.010, 0.019)	0.526	0.640	0.991 (0.923, 1.063)	0.800	0.837
Free cholesterol to total lipids ratio in large LDL	4,778	-0.002 (-0.016, 0.012)	0.797	0.867	0.923 (0.855, 0.997)	0.042	0.082
Free cholesterol to total lipids ratio in large VLDL	4,778	-0.007 (-0.022, 0.007)	0.325	0.475	0.968 (0.892, 1.049)	0.425	0.532
Free cholesterol to total lipids ratio in medium HDL	4,778	0.006 (-0.009, 0.020)	0.421	0.557	1.054 (0.983, 1.130)	0.142	0.209
Free cholesterol to total lipids ratio in medium LDL	4,778	-0.007 (-0.021, 0.007)	0.339	0.483	0.933 (0.860, 1.013)	0.098	0.155
Free cholesterol to total lipids ratio in medium VLDL	4,778	-0.019 (-0.034, -0.005)	0.008	0.043	0.879 (0.811, 0.954)	0.002	0.007
Free cholesterol to total lipids ratio in small HDL	4,778	0.004 (-0.011, 0.018)	0.610	0.708	1.062 (0.991, 1.138)	0.087	0.142
Free cholesterol to total lipids ratio in small LDL	4,778	0.006 (-0.008, 0.020)	0.407	0.555	0.912 (0.845, 0.984)	0.017	0.041
Free cholesterol to total lipids ratio in small VLDL	4,778	-0.015 (-0.029, -0.001)	0.039	0.126	0.910 (0.845, 0.980)	0.013	0.033
Free cholesterol to total lipids ratio in very large HDL	4,778	0.008 (-0.007, 0.022)	0.302	0.450	1.102 (1.026, 1.183)	0.008	0.022
Free cholesterol to total lipids ratio in very large VLDL	4,778	0.003 (-0.011, 0.016)	0.700	0.776	0.912 (0.844, 0.986)	0.021	0.045
Free cholesterol to total lipids ratio in very small VLDL	4,778	-0.013 (-0.027, 0.001)	0.065	0.175	0.901 (0.836, 0.971)	0.006	0.020
Phospholipids to total lipds ratio in IDL	4,778	-0.004 (-0.018, 0.010)	0.602	0.709	0.976 (0.906, 1.053)	0.533	0.625
Phospholipids to total lipds ratio in chylomicrons and extremely large VLDL	4,778	0.025 (0.011, 0.039)	0.001	0.006	0.968 (0.900, 1.040)	0.373	0.480
Phospholipids to total lipds ratio in large HDL	4,778	0.010 (-0.005, 0.024)	0.188	0.306	1.038 (0.968, 1.113)	0.298	0.393
Phospholipids to total lipds ratio in large LDL	4,778	-0.005 (-0.020, 0.009)	0.470	0.601	0.991 (0.916, 1.071)	0.817	0.851
Phospholipids to total lipds ratio in large VLDL	4,778	-0.022 (-0.036, -0.008)	0.003	0.017	0.897 (0.829, 0.969)	0.006	0.019
Phospholipids to total lipds ratio in medium HDL	4,778	0.011 (-0.004, 0.026)	0.138	0.254	1.050 (0.979, 1.126)	0.171	0.237
Phospholipids to total lipds ratio in medium LDL	4,778	0.001 (-0.013, 0.015)	0.894	0.927	1.140 (1.056, 1.232)	0.001	0.004
Phospholipids to total lipds ratio in medium VLDL	4,778	-0.018 (-0.032, -0.004)	0.014	0.064	0.924 (0.860, 0.993)	0.032	0.065
Phospholipids to total lipds ratio in small HDL	4,778	0.006 (-0.008, 0.021)	0.379	0.530	1.087 (1.014, 1.165)	0.019	0.042
Phospholipids to total lipds ratio in small LDL	4,778	-0.003 (-0.017, 0.010)	0.646	0.724	1.061 (0.978, 1.150)	0.154	0.220
Phospholipids to total lipds ratio in small VLDL	4,778	-0.006 (-0.020, 0.008)	0.402	0.552	0.966 (0.899, 1.039)	0.352	0.455
Phospholipids to total lipds ratio in very large HDL	4,778	0.001 (-0.013, 0.016)	0.854	0.911	0.973 (0.905, 1.045)	0.451	0.561
Phospholipids to total lipds ratio in very large VLDL	4,778	0.002 (-0.012, 0.016)	0.816	0.874	0.937 (0.870, 1.009)	0.086	0.142
Phospholipids to total lipds ratio in very small VLDL	4,778	-0.008 (-0.022, 0.006)	0.270	0.414	0.948 (0.882, 1.019)	0.149	0.214
Total cholesterol to total lipids ratio in IDL	4,778	-0.007 (-0.021, 0.007)	0.309	0.454	0.851 (0.790, 0.916)	<0.001	0.001
Total cholesterol to total lipids ratio in chylomicrons and extremely large VLDL	4,778	-0.016 (-0.030, -0.002)	0.022	0.087	0.878 (0.811, 0.949)	0.001	0.005
Total cholesterol to total lipids ratio in large HDL	4,778	0.010 (-0.004, 0.025)	0.170	0.284	1.026 (0.957, 1.100)	0.467	0.574
Total cholesterol to total lipids ratio in large LDL	4,778	0.007 (-0.007, 0.021)	0.332	0.479	1.011 (0.936, 1.091)	0.789	0.833
Total cholesterol to total lipids ratio in large VLDL	4,778	0.015 (0.001, 0.029)	0.037	0.123	1.012 (0.937, 1.094)	0.759	0.814
Total cholesterol to total lipids ratio in medium HDL	4,778	0.010 (-0.005, 0.024)	0.190	0.307	1.057 (0.985, 1.134)	0.121	0.185
Total cholesterol to total lipids ratio in medium LDL	4,778	-0.004 (-0.018, 0.010)	0.612	0.706	0.876 (0.811, 0.945)	0.001	0.004
Total cholesterol to total lipids ratio in medium VLDL	4,778	0.005 (-0.010, 0.019)	0.519	0.635	1.058 (0.976, 1.146)	0.170	0.239
Total cholesterol to total lipids ratio in small HDL	4,778	0.000 (-0.014, 0.015)	0.985	0.994	1.013 (0.946, 1.085)	0.715	0.788
Total cholesterol to total lipids ratio in small LDL	4,778	-0.003 (-0.018, 0.011)	0.629	0.715	0.927 (0.856, 1.003)	0.060	0.107
Total cholesterol to total lipids ratio in small VLDL	4,778	0.006 (-0.008, 0.020)	0.413	0.554	1.032 (0.958, 1.111)	0.405	0.511
Total cholesterol to total lipids ratio in very large HDL	4,778	0.008 (-0.006, 0.022)	0.245	0.386	0.987 (0.916, 1.064)	0.737	0.808
Total cholesterol to total lipids ratio in very large VLDL	4,778	-0.004 (-0.018, 0.011)	0.618	0.710	1.026 (0.953, 1.104)	0.500	0.598
Total cholesterol to total lipids ratio in very small VLDL	4,778	0.004 (-0.010, 0.018)	0.605	0.706	1.025 (0.952, 1.103)	0.514	0.606
Triglycerides to total lipids ratio in IDL	4,778	0.000 (-0.014, 0.014)	0.950	0.972	0.983 (0.913, 1.059)	0.653	0.739
Triglycerides to total lipids ratio in chylomicrons and extremely large VLDL	4,778	0.015 (0.001, 0.028)	0.036	0.121	1.156 (1.071, 1.247)	<0.001	0.003

Triglycerides to total lipids ratio in large HDL	4,778	0.007 (-0.008, 0.021)	0.374	0.527	1.010 (0.942, 1.084)	0.779	0.831
Triglycerides to total lipids ratio in large LDL	4,778	-0.003 (-0.017, 0.011)	0.644	0.725	0.926 (0.856, 1.003)	0.058	0.106
Triglycerides to total lipids ratio in large VLDL	4,778	-0.013 (-0.027, 0.000)	0.057	0.166	1.044 (0.968, 1.125)	0.266	0.356
Triglycerides to total lipids ratio in medium HDL	4,778	0.011 (-0.004, 0.026)	0.141	0.256	1.034 (0.965, 1.109)	0.341	0.444
Triglycerides to total lipids ratio in medium LDL	4,778	0.001 (-0.013, 0.015)	0.891	0.928	1.069 (0.992, 1.153)	0.081	0.136
Triglycerides to total lipids ratio in medium VLDL	4,778	-0.013 (-0.026, 0.001)	0.073	0.184	1.016 (0.942, 1.097)	0.677	0.758
Triglycerides to total lipids ratio in small HDL	4,778	-0.001 (-0.015, 0.013)	0.879	0.920	0.986 (0.918, 1.060)	0.710	0.787
Triglycerides to total lipids ratio in small LDL	4,778	0.001 (-0.014, 0.015)	0.912	0.937	1.086 (1.010, 1.167)	0.025	0.052
Triglycerides to total lipids ratio in small VLDL	4,778	-0.010 (-0.024, 0.004)	0.163	0.277	1.026 (0.951, 1.106)	0.505	0.601
Triglycerides to total lipids ratio in very large HDL	4,778	-0.007 (-0.021, 0.007)	0.301	0.452	1.016 (0.942, 1.096)	0.676	0.760
Triglycerides to total lipids ratio in very large VLDL	4,778	0.000 (-0.014, 0.014)	0.986	0.991	1.096 (1.019, 1.179)	0.013	0.033
Triglycerides to total lipids ratio in very small VLDL	4,778	-0.007 (-0.021, 0.007)	0.330	0.479	1.063 (0.988, 1.143)	0.102	0.160
Total cholines	4,778	-0.003 (-0.017, 0.011)	0.643	0.727	1.059 (0.984, 1.140)	0.127	0.190
Phosphatidylcholine and other cholines	4,778	0.000 (-0.014, 0.014)	0.975	0.988	1.096 (1.019, 1.178)	0.013	0.033
Total phosphoglycerides	4,778	0.006 (-0.008, 0.021)	0.410	0.555	0.952 (0.886, 1.022)	0.176	0.244
Ratio of triglycerides to phosphoglycerides	4,778	-0.009 (-0.023, 0.006)	0.232	0.368	1.049 (0.974, 1.129)	0.207	0.280
Albumin	4,777	0.002 (-0.013, 0.016)	0.806	0.867	1.122 (1.044, 1.206)	0.002	0.007
Creatinine	4,778	0.007 (-0.007, 0.021)	0.306	0.453	0.876 (0.811, 0.947)	0.001	0.004
Estimated degree of unsaturation	4,746	0.006 (-0.006, 0.018)	0.356	0.503	1.086 (0.984, 1.199)	0.102	0.161
Sphingomyelins	4,778	0.005 (-0.009, 0.018)	0.502	0.628	0.972 (0.902, 1.047)	0.458	0.566

Models were adjusted for age, sex, region, education, household income, occupation, marital status, tea drinking habit, smoking status, alcohol intake, physical activity, self-rated health, fasting time, and frequency of other 11 food groups. Sample size of analyses (n) of some metabolic markers involved was less than 4,778, since results of these metabolic markers were rejected by the quality control process among some participants.
