# Supplementary material for: Association of Red Meat Consumption, Metabolic Markers, and Risk of Cardiovascular Diseases
Source: Front Nutr. 2022 Apr 15;9:833271. doi: 10.3389/fnut.2022.833271 (PMC9051033; doi:10.3389/fnut.2022.833271)
Supplement: Supplementary file 3 [file Table_3.PDF]

Table S3. ORs (95% CI) for MI, IS, and ICH per SD higher log-transformed metabolic markers.

Metabolic marker	OR (95% CI) for MI	*p*	FDR-*p*	OR (95% CI) for IS	*p*	FDR-*p*	OR (95% CI) for ICH	*p*	FDR-*p*
Mean diameter for VLDL particles	1.099 (0.999, 1.208)	0.052	0.084	1.112 (1.018, 1.214)	0.018	0.064	1.042 (0.959, 1.132)	0.332	1.245
Mean diameter for LDL particles	0.894 (0.809, 0.989)	0.030	0.055	0.836 (0.757, 0.922)	<0.001	0.007	0.954 (0.876, 1.038)	0.275	1.585
Mean diameter for HDL particles	0.856 (0.777, 0.944)	0.002	0.005	0.914 (0.833, 1.003)	0.058	0.132	0.981 (0.903, 1.065)	0.643	0.959
Concentration of chylomicrons and extremely large VLDL particles	1.102 (0.998, 1.215)	0.054	0.086	1.085 (0.997, 1.180)	0.060	0.133	1.024 (0.944, 1.110)	0.570	1.025
Concentration of very large VLDL particles	1.121 (1.019, 1.233)	0.019	0.037	1.116 (1.022, 1.218)	0.015	0.055	1.032 (0.950, 1.121)	0.461	1.093
Concentration of large VLDL particles	1.174 (1.067, 1.292)	0.001	0.003	1.130 (1.035, 1.234)	0.006	0.032	1.049 (0.965, 1.140)	0.263	1.852
Concentration of medium VLDL particles	1.196 (1.088, 1.316)	<0.001	0.001	1.148 (1.050, 1.255)	0.002	0.020	1.047 (0.962, 1.138)	0.288	1.410
Concentration of small VLDL particles	1.246 (1.132, 1.371)	<0.001	<0.001	1.157 (1.056, 1.267)	0.002	0.017	1.044 (0.960, 1.136)	0.313	1.282
Concentration of very small VLDL particles	1.247 (1.132, 1.374)	<0.001	<0.001	1.128 (1.026, 1.239)	0.013	0.051	1.015 (0.932, 1.105)	0.734	0.966
Concentration of IDL particles	1.169 (1.060, 1.289)	0.002	0.005	1.092 (0.994, 1.201)	0.067	0.147	0.989 (0.908, 1.077)	0.799	0.967
Concentration of large LDL particles	1.177 (1.067, 1.298)	0.001	0.004	1.108 (1.008, 1.217)	0.033	0.093	1.000 (0.919, 1.089)	0.996	1.001
Concentration of medium LDL particles	1.183 (1.073, 1.304)	0.001	0.003	1.132 (1.030, 1.243)	0.010	0.045	1.014 (0.931, 1.104)	0.755	0.976
Concentration of small LDL particles	1.195 (1.084, 1.318)	<0.001	0.002	1.153 (1.049, 1.268)	0.003	0.021	1.017 (0.934, 1.107)	0.697	0.963
Concentration of very large HDL particles	0.940 (0.855, 1.034)	0.205	0.272	0.976 (0.892, 1.068)	0.601	0.705	0.999 (0.921, 1.084)	0.985	1.003
Concentration of large HDL particles	0.836 (0.758, 0.923)	<0.001	0.002	0.908 (0.826, 0.997)	0.044	0.110	0.978 (0.897, 1.067)	0.617	0.984
Concentration of medium HDL particles	0.872 (0.791, 0.961)	0.006	0.013	0.988 (0.897, 1.088)	0.804	0.854	0.968 (0.889, 1.055)	0.462	1.083
Concentration of small HDL particles	1.020 (0.926, 1.124)	0.685	0.755	1.068 (0.971, 1.175)	0.177	0.291	1.005 (0.924, 1.092)	0.916	0.986
Total lipids in chylomicrons and extremely large VLDL	1.103 (1.000, 1.217)	0.049	0.081	1.086 (0.998, 1.182)	0.056	0.132	1.024 (0.945, 1.110)	0.566	1.027
Total lipids in very large VLDL	1.121 (1.019, 1.233)	0.019	0.036	1.116 (1.022, 1.218)	0.014	0.056	1.031 (0.949, 1.120)	0.467	1.083
Total lipids in large VLDL	1.173 (1.066, 1.291)	0.001	0.004	1.129 (1.034, 1.232)	0.007	0.033	1.048 (0.964, 1.139)	0.274	1.665
Total lipids in medium VLDL	1.196 (1.088, 1.316)	<0.001	0.001	1.147 (1.049, 1.254)	0.003	0.020	1.046 (0.962, 1.137)	0.295	1.326
Total lipids in small VLDL	1.250 (1.136, 1.376)	<0.001	<0.001	1.159 (1.057, 1.269)	0.002	0.017	1.044 (0.960, 1.136)	0.316	1.226
Total lipids in very small VLDL	1.235 (1.120, 1.360)	<0.001	<0.001	1.119 (1.018, 1.229)	0.020	0.065	1.011 (0.929, 1.102)	0.795	0.966
Total lipids in IDL	1.156 (1.048, 1.275)	0.004	0.009	1.089 (0.991, 1.197)	0.078	0.159	0.987 (0.906, 1.075)	0.759	0.975
Total lipids in large LDL	1.167 (1.058, 1.287)	0.002	0.005	1.104 (1.005, 1.213)	0.039	0.104	0.999 (0.917, 1.087)	0.976	1.012
Total lipids in medium LDL	1.177 (1.068, 1.298)	0.001	0.003	1.129 (1.028, 1.241)	0.011	0.048	1.013 (0.930, 1.103)	0.774	0.979
Total lipids in small LDL	1.187 (1.077, 1.308)	0.001	0.003	1.146 (1.043, 1.260)	0.005	0.026	1.015 (0.932, 1.106)	0.729	0.970
Total lipids in very large HDL	0.942 (0.856, 1.035)	0.214	0.283	0.978 (0.893, 1.070)	0.621	0.720	0.999 (0.920, 1.084)	0.977	1.009
Total lipids in large HDL	0.835 (0.756, 0.921)	<0.001	0.002	0.907 (0.826, 0.996)	0.042	0.107	0.977 (0.896, 1.066)	0.603	0.984
Total lipids in medium HDL	0.867 (0.787, 0.956)	0.004	0.010	0.987 (0.896, 1.086)	0.784	0.836	0.969 (0.890, 1.056)	0.478	1.075
Total lipids in small HDL	1.015 (0.922, 1.119)	0.758	0.800	1.063 (0.966, 1.169)	0.212	0.338	1.003 (0.922, 1.090)	0.950	1.004
Total cholesterol	1.154 (1.048, 1.271)	0.004	0.009	1.127 (1.025, 1.239)	0.014	0.055	1.002 (0.920, 1.092)	0.959	1.004
Total cholesterol in VLDL	1.228 (1.115, 1.354)	<0.001	<0.001	1.169 (1.063, 1.286)	0.001	0.017	1.018 (0.935, 1.110)	0.678	0.966
Total cholesterol in chylomicrons and extremely large VLDL	1.231 (1.119, 1.355)	<0.001	<0.001	1.167 (1.064, 1.280)	0.001	0.015	1.036 (0.952, 1.128)	0.413	1.208
Total cholesterol in very large VLDL	1.113 (1.008, 1.229)	0.034	0.059	1.081 (0.994, 1.176)	0.070	0.149	1.024 (0.945, 1.110)	0.556	1.025
Total cholesterol in large VLDL	1.097 (0.997, 1.208)	0.058	0.091	1.096 (1.005, 1.196)	0.038	0.102	1.032 (0.950, 1.120)	0.459	1.099
Total cholesterol in medium VLDL	1.157 (1.050, 1.275)	0.003	0.008	1.104 (1.013, 1.204)	0.025	0.080	1.039 (0.956, 1.129)	0.366	1.212
Total cholesterol in small VLDL	1.189 (1.081, 1.309)	<0.001	0.002	1.131 (1.035, 1.236)	0.007	0.033	1.037 (0.953, 1.128)	0.398	1.210
Total cholesterol in very small VLDL	1.250 (1.135, 1.377)	<0.001	<0.001	1.152 (1.049, 1.266)	0.003	0.021	1.040 (0.955, 1.132)	0.365	1.227
Total cholesterol in IDL	1.176 (1.065, 1.298)	0.001	0.004	1.080 (0.983, 1.187)	0.110	0.209	0.997 (0.916, 1.086)	0.951	1.000
Total cholesterol in LDL	1.128 (1.023, 1.245)	0.016	0.031	1.083 (0.986, 1.190)	0.096	0.191	0.982 (0.902, 1.069)	0.677	0.970
Total cholesterol in large LDL	1.144 (1.036, 1.263)	0.008	0.018	1.120 (1.016, 1.234)	0.023	0.074	0.992 (0.915, 1.076)	0.847	0.972
Total cholesterol in medium LDL	1.145 (1.037, 1.264)	0.007	0.016	1.102 (1.002, 1.212)	0.046	0.113	0.993 (0.914, 1.079)	0.868	0.972
Total cholesterol in small LDL	1.133 (1.029, 1.248)	0.011	0.024	1.109 (1.010, 1.218)	0.030	0.091	1.009 (0.927, 1.099)	0.838	0.967
Total cholesterol in HDL	1.128 (1.025, 1.242)	0.014	0.028	1.110 (1.011, 1.219)	0.028	0.087	1.010 (0.927, 1.099)	0.824	0.965
Total cholesterol in HDL2	0.864 (0.785, 0.950)	0.003	0.007	0.956 (0.871, 1.050)	0.350	0.487	0.977 (0.898, 1.064)	0.599	0.984
Total cholesterol in HDL3	0.842 (0.766, 0.925)	<0.001	0.002	0.948 (0.863, 1.041)	0.260	0.391	0.968 (0.889, 1.054)	0.450	1.100
Total cholesterol in very large HDL	1.098 (0.997, 1.208)	0.057	0.090	1.067 (0.973, 1.171)	0.170	0.285	1.060 (0.976, 1.153)	0.168	2.229
Total cholesterol in large HDL	0.995 (0.906, 1.092)	0.910	0.918	1.019 (0.931, 1.114)	0.689	0.767	1.011 (0.932, 1.097)	0.790	0.971
Total cholesterol in medium HDL	0.825 (0.748, 0.910)	<0.001	0.001	0.903 (0.822, 0.992)	0.033	0.093	0.972 (0.892, 1.060)	0.525	1.046
Total cholesterol in small HDL	0.848 (0.771, 0.933)	0.001	0.003	0.989 (0.899, 1.088)	0.819	0.857	0.967 (0.890, 1.051)	0.436	1.140
Remnant cholesterol (non-HDL, non-LDL -cholesterol)	1.040 (0.945, 1.145)	0.426	0.518	1.041 (0.951, 1.139)	0.388	0.523	1.008 (0.926, 1.097)	0.856	0.972
Esterified cholesterol	0.971 (0.897, 1.052)	0.470	0.556	0.995 (0.841, 1.178)	0.958	0.966	0.936 (0.696, 1.259)	0.662	0.967
Cholesterol esters in chylomicrons and extremely large VLDL	1.114 (1.009, 1.229)	0.033	0.058	1.080 (0.992, 1.175)	0.076	0.157	1.022 (0.943, 1.108)	0.597	0.995
Cholesterol esters in very large VLDL	1.088 (0.989, 1.198)	0.084	0.128	1.084 (0.994, 1.182)	0.068	0.148	1.033 (0.951, 1.122)	0.442	1.131
Cholesterol esters in large VLDL	1.135 (1.025, 1.257)	0.015	0.030	1.063 (0.979, 1.155)	0.147	0.253	1.047 (0.963, 1.138)	0.279	1.572
Cholesterol esters in medium VLDL	1.169 (1.061, 1.287)	0.002	0.005	1.114 (1.019, 1.219)	0.018	0.063	1.021 (0.938, 1.111)	0.635	0.965
Cholesterol esters in small VLDL	1.212 (1.100, 1.337)	<0.001	0.001	1.133 (1.030, 1.246)	0.010	0.046	1.029 (0.945, 1.121)	0.504	1.069
Cholesterol esters in very small VLDL	1.161 (1.051, 1.282)	0.003	0.008	1.078 (0.980, 1.186)	0.122	0.231	0.993 (0.912, 1.082)	0.878	0.978
Cholesterol esters in IDL	1.145 (1.038, 1.264)	0.007	0.016	1.101 (1.002, 1.210)	0.046	0.113	0.983 (0.902, 1.071)	0.696	0.967
Cholesterol esters in large LDL	1.173 (1.051, 1.309)	0.004	0.010	1.116 (1.008, 1.235)	0.034	0.093	0.989 (0.916, 1.068)	0.776	0.975
Cholesterol esters in medium LDL	1.084 (0.985, 1.193)	0.099	0.145	1.089 (0.990, 1.197)	0.079	0.159	1.004 (0.926, 1.089)	0.916	0.982
Cholesterol esters in small LDL	1.071 (0.975, 1.177)	0.154	0.210	1.081 (0.983, 1.189)	0.106	0.205	1.004 (0.926, 1.088)	0.921	0.982
Cholesterol esters in very large HDL	1.017 (0.926, 1.116)	0.730	0.786	1.036 (0.947, 1.133)	0.440	0.569	1.020 (0.941, 1.106)	0.633	0.968
Cholesterol esters in large HDL	0.823 (0.746, 0.908)	<0.001	0.001	0.902 (0.821, 0.991)	0.031	0.092	0.973 (0.892, 1.061)	0.537	1.043
Cholesterol esters in medium HDL	0.847 (0.770, 0.931)	0.001	0.003	0.993 (0.903, 1.092)	0.888	0.912	0.966 (0.889, 1.049)	0.410	1.229
Cholesterol esters in small HDL	1.024 (0.924, 1.134)	0.650	0.728	1.029 (0.941, 1.126)	0.527	0.638	0.985 (0.908, 1.070)	0.725	0.971
Free cholesterol	1.117 (1.017, 1.227)	0.021	0.039	1.160 (1.053, 1.278)	0.003	0.019	0.988 (0.906, 1.077)	0.783	0.979
Free cholesterol in chylomicrons and extremely large VLDL	1.093 (0.992, 1.205)	0.072	0.112	1.089 (1.001, 1.186)	0.047	0.115	1.028 (0.948, 1.114)	0.502	1.076
Free cholesterol in very large VLDL	1.077 (0.979, 1.186)	0.129	0.183	1.097 (1.007, 1.196)	0.035	0.095	1.027 (0.947, 1.115)	0.520	1.045
Free cholesterol in large VLDL	1.156 (1.048, 1.274)	0.004	0.009	1.111 (1.020, 1.210)	0.016	0.056	1.053 (0.969, 1.143)	0.223	2.276
Free cholesterol in medium VLDL	1.199 (1.089, 1.319)	<0.001	0.001	1.139 (1.043, 1.244)	0.004	0.023	1.050 (0.966, 1.142)	0.249	2.239
Free cholesterol in small VLDL	1.259 (1.144, 1.385)	<0.001	<0.001	1.152 (1.051, 1.262)	0.002	0.021	1.045 (0.961, 1.136)	0.305	1.271
Free cholesterol in very small VLDL	1.193 (1.079, 1.320)	0.001	0.003	1.073 (0.979, 1.175)	0.131	0.240	1.004 (0.925, 1.089)	0.930	0.987
Free cholesterol in IDL	1.081 (0.980, 1.192)	0.119	0.172	1.039 (0.947, 1.139)	0.421	0.557	0.977 (0.900, 1.061)	0.583	1.008
Free cholesterol in large LDL	1.109 (1.005, 1.222)	0.039	0.067	1.059 (0.965, 1.163)	0.226	0.350	0.983 (0.904, 1.069)	0.695	0.971
Free cholesterol in medium LDL	1.175 (1.066, 1.295)	0.001	0.004	1.119 (1.018, 1.230)	0.019	0.065	1.005 (0.923, 1.096)	0.901	0.993
Free cholesterol in small LDL	1.181 (1.072, 1.301)	0.001	0.003	1.130 (1.028, 1.242)	0.012	0.048	1.012 (0.929, 1.101)	0.790	0.966
Free cholesterol in very large HDL	0.943 (0.859, 1.036)	0.223	0.292	0.978 (0.893, 1.070)	0.625	0.721	0.991 (0.913, 1.076)	0.826	0.958
Free cholesterol in large HDL	0.844 (0.766, 0.931)	0.001	0.003	0.914 (0.833, 1.003)	0.059	0.132	0.971 (0.893, 1.055)	0.486	1.062
Free cholesterol in medium HDL	0.862 (0.784, 0.949)	0.002	0.006	0.973 (0.885, 1.071)	0.578	0.688	0.975 (0.895, 1.061)	0.555	1.041
Free cholesterol in small HDL	0.953 (0.865, 1.050)	0.333	0.410	1.021 (0.927, 1.125)	0.675	0.756	0.999 (0.919, 1.086)	0.983	1.005

Total triglycerides	1.210 (1.101, 1.331)	<0.001	0.001	1.147 (1.049, 1.253)	0.003	0.019	1.051 (0.966, 1.143)	0.249	2.154
Triglycerides in VLDL	1.193 (1.085, 1.312)	<0.001	0.002	1.150 (1.051, 1.257)	0.002	0.020	1.049 (0.964, 1.140)	0.266	1.712
Triglycerides in chylomicrons and extremely large VLDL	1.093 (0.991, 1.207)	0.076	0.118	1.072 (0.987, 1.166)	0.100	0.196	1.023 (0.945, 1.109)	0.570	1.017
Triglycerides in very large VLDL	1.120 (1.018, 1.232)	0.020	0.037	1.116 (1.022, 1.218)	0.015	0.055	1.033 (0.950, 1.122)	0.449	1.123
Triglycerides in large VLDL	1.176 (1.069, 1.294)	0.001	0.003	1.135 (1.039, 1.239)	0.005	0.027	1.053 (0.968, 1.144)	0.228	2.229
Triglycerides in medium VLDL	1.195 (1.087, 1.315)	<0.001	0.001	1.150 (1.052, 1.257)	0.002	0.020	1.050 (0.966, 1.142)	0.254	2.114
Triglycerides in small VLDL	1.226 (1.114, 1.349)	<0.001	<0.001	1.144 (1.046, 1.251)	0.003	0.021	1.047 (0.963, 1.139)	0.281	1.503
Triglycerides in very small VLDL	1.258 (1.144, 1.384)	<0.001	<0.001	1.124 (1.026, 1.230)	0.012	0.048	1.033 (0.950, 1.124)	0.448	1.134
Triglycerides in IDL	1.275 (1.158, 1.405)	<0.001	<0.001	1.081 (0.986, 1.185)	0.098	0.193	1.025 (0.944, 1.113)	0.560	1.024
Triglycerides in LDL	1.273 (1.155, 1.403)	<0.001	<0.001	1.091 (0.995, 1.196)	0.065	0.144	1.037 (0.954, 1.127)	0.396	1.221
Triglycerides in large LDL	1.276 (1.156, 1.409)	<0.001	<0.001	1.070 (0.975, 1.174)	0.155	0.263	1.023 (0.942, 1.110)	0.597	1.002
Triglycerides in medium LDL	1.261 (1.144, 1.389)	<0.001	<0.001	1.108 (1.010, 1.215)	0.030	0.090	1.039 (0.959, 1.127)	0.351	1.216
Triglycerides in small LDL	1.274 (1.157, 1.403)	<0.001	<0.001	1.168 (1.065, 1.280)	0.001	0.014	1.049 (0.966, 1.138)	0.255	2.051
Triglycerides in HDL	1.120 (1.016, 1.235)	0.023	0.042	1.042 (0.951, 1.141)	0.379	0.514	1.026 (0.942, 1.118)	0.553	1.045
Triglycerides in very large HDL	1.129 (1.021, 1.249)	0.018	0.036	1.051 (0.960, 1.151)	0.281	0.405	1.048 (0.962, 1.142)	0.280	1.537
Triglycerides in large HDL	1.013 (0.909, 1.128)	0.820	0.854	0.939 (0.855, 1.032)	0.194	0.316	1.022 (0.936, 1.117)	0.624	0.982
Triglycerides in medium HDL	1.082 (0.984, 1.189)	0.104	0.151	1.058 (0.967, 1.158)	0.219	0.345	0.985 (0.905, 1.072)	0.723	0.974
Triglycerides in small HDL	1.174 (1.068, 1.291)	0.001	0.003	1.085 (0.992, 1.186)	0.073	0.154	1.018 (0.938, 1.106)	0.664	0.964
Phospholipids in chylomicrons and extremely large VLDL	1.047 (0.954, 1.149)	0.338	0.413	1.099 (0.997, 1.211)	0.057	0.132	0.987 (0.905, 1.077)	0.773	0.983
Phospholipids in very large VLDL	1.098 (1.000, 1.206)	0.050	0.081	1.158 (1.052, 1.275)	0.003	0.019	1.040 (0.957, 1.131)	0.355	1.209
Phospholipids in large VLDL	1.083 (0.987, 1.189)	0.090	0.136	1.156 (1.049, 1.274)	0.004	0.022	1.038 (0.955, 1.128)	0.379	1.200
Phospholipids in medium VLDL	1.056 (0.964, 1.158)	0.242	0.315	1.122 (1.019, 1.236)	0.019	0.065	1.012 (0.932, 1.100)	0.770	0.985
Phospholipids in small VLDL	1.094 (0.992, 1.206)	0.071	0.111	1.090 (1.001, 1.186)	0.048	0.114	1.023 (0.944, 1.108)	0.579	1.018
Phospholipids in very small VLDL	1.088 (0.988, 1.198)	0.086	0.130	1.095 (1.004, 1.194)	0.040	0.105	1.027 (0.947, 1.115)	0.518	1.050
Phospholipids in IDL	1.176 (1.068, 1.295)	0.001	0.003	1.128 (1.033, 1.231)	0.007	0.033	1.049 (0.965, 1.140)	0.261	2.025
Phospholipids in large LDL	1.200 (1.091, 1.320)	<0.001	0.001	1.148 (1.050, 1.255)	0.002	0.021	1.044 (0.960, 1.135)	0.315	1.266
Phospholipids in medium LDL	1.236 (1.123, 1.360)	<0.001	<0.001	1.144 (1.044, 1.253)	0.004	0.024	1.031 (0.948, 1.121)	0.478	1.066
Phospholipids in small LDL	1.181 (1.070, 1.304)	0.001	0.003	1.095 (0.997, 1.202)	0.057	0.133	0.995 (0.916, 1.082)	0.912	0.992
Phospholipids in very large HDL	1.130 (1.025, 1.245)	0.014	0.028	1.067 (0.972, 1.172)	0.171	0.284	0.983 (0.902, 1.071)	0.689	0.969
Phospholipids in large HDL	1.169 (1.061, 1.289)	0.002	0.005	1.113 (1.012, 1.223)	0.028	0.087	0.992 (0.910, 1.082)	0.864	0.972
Phospholipids in medium HDL	1.220 (1.106, 1.345)	<0.001	0.001	1.166 (1.059, 1.284)	0.002	0.017	1.005 (0.922, 1.095)	0.905	0.994
Phospholipids in small HDL	1.214 (1.102, 1.339)	<0.001	0.001	1.176 (1.067, 1.295)	0.001	0.014	1.008 (0.926, 1.098)	0.853	0.975
Apolipoprotein A-I	0.892 (0.811, 0.981)	0.019	0.036	0.943 (0.861, 1.033)	0.205	0.332	0.985 (0.907, 1.070)	0.718	0.973
Apolipoprotein B	0.844 (0.765, 0.931)	0.001	0.003	0.918 (0.836, 1.008)	0.074	0.154	0.979 (0.899, 1.068)	0.636	0.961
Ratio of apolipoprotein B to apolipoprotein A-I	0.883 (0.801, 0.974)	0.013	0.028	0.978 (0.888, 1.077)	0.655	0.744	0.979 (0.897, 1.068)	0.629	0.975
Total fatty acids	0.948 (0.860, 1.043)	0.273	0.350	1.028 (0.935, 1.130)	0.572	0.684	0.990 (0.911, 1.075)	0.806	0.960
Saturated fatty acids	0.967 (0.878, 1.065)	0.493	0.581	1.039 (0.945, 1.143)	0.429	0.558	1.011 (0.929, 1.100)	0.801	0.964
Monounsaturated fatty acids; 16:1, 18:1	1.261 (1.145, 1.389)	<0.001	<0.001	1.209 (1.099, 1.332)	<0.001	0.004	1.032 (0.946, 1.125)	0.483	1.066
Polyunsaturated fatty acids	1.276 (1.158, 1.406)	<0.001	<0.001	1.183 (1.075, 1.303)	0.001	0.009	1.027 (0.941, 1.120)	0.555	1.033
Omega-3 fatty acids	1.161 (1.057, 1.276)	0.002	0.005	1.234 (1.121, 1.358)	<0.001	0.001	1.035 (0.951, 1.127)	0.426	1.142
22:6, docosahexaenoic acid	1.143 (1.042, 1.254)	0.005	0.011	1.202 (1.094, 1.322)	<0.001	0.004	1.024 (0.940, 1.115)	0.588	1.010
Omega-6 fatty acids	1.188 (1.082, 1.305)	<0.001	0.002	1.195 (1.089, 1.313)	<0.001	0.005	1.042 (0.958, 1.134)	0.339	1.229
18:2, linoleic acid	1.129 (1.026, 1.242)	0.013	0.027	1.286 (1.162, 1.425)	<0.001	<0.001	1.037 (0.950, 1.131)	0.416	1.200
Ratio of saturated fatty acids to total fatty acids	1.165 (1.058, 1.283)	0.002	0.005	1.208 (1.093, 1.334)	<0.001	0.005	0.980 (0.898, 1.070)	0.648	0.959
Ratio of monounsaturated fatty acids to total fatty acids	1.204 (1.096, 1.322)	<0.001	0.001	1.173 (1.063, 1.294)	0.002	0.018	0.995 (0.913, 1.084)	0.906	0.990
Ratio of polyunsaturated fatty acids to total fatty acids	1.116 (1.015, 1.227)	0.023	0.042	1.289 (1.163, 1.428)	<0.001	<0.001	1.047 (0.960, 1.141)	0.300	1.298
Ratio of omega-3 fatty acids to total fatty acids	0.950 (0.866, 1.043)	0.284	0.358	1.153 (0.952, 1.396)	0.146	0.253	0.980 (0.898, 1.069)	0.642	0.963
Ratio of 22:6 docosahexaenoic acid to total fatty acids	0.908 (0.826, 0.998)	0.045	0.075	0.877 (0.799, 0.962)	0.005	0.028	0.970 (0.883, 1.064)	0.516	1.065
Ratio of omega-6 fatty acids to total fatty acids	1.154 (1.049, 1.270)	0.003	0.008	1.007 (0.918, 1.105)	0.877	0.905	1.050 (0.964, 1.144)	0.263	1.911
Ratio of 18:2 linoleic acid to total fatty acids	0.934 (0.848, 1.028)	0.163	0.221	1.059 (0.965, 1.163)	0.223	0.349	1.020 (0.934, 1.115)	0.657	0.966
Alanine	1.054 (0.957, 1.160)	0.284	0.358	1.022 (0.927, 1.128)	0.661	0.744	0.929 (0.850, 1.015)	0.101	1.902
Glutamine	1.049 (0.960, 1.147)	0.287	0.359	0.935 (0.856, 1.021)	0.134	0.243	0.966 (0.888, 1.051)	0.423	1.146
Histidine	0.927 (0.844, 1.018)	0.114	0.166	1.048 (0.958, 1.146)	0.310	0.439	1.048 (0.960, 1.145)	0.293	1.347
Isoleucine	0.936 (0.856, 1.024)	0.149	0.206	0.984 (0.900, 1.077)	0.730	0.797	1.013 (0.924, 1.110)	0.786	0.977
Leucine	1.014 (0.922, 1.116)	0.772	0.812	1.022 (0.937, 1.115)	0.617	0.719	0.972 (0.896, 1.055)	0.499	1.080
Phenylalanine	0.905 (0.820, 0.998)	0.045	0.075	0.967 (0.880, 1.062)	0.482	0.602	0.970 (0.892, 1.054)	0.467	1.073
Tyrosine	0.978 (0.890, 1.075)	0.651	0.725	1.032 (0.947, 1.126)	0.470	0.591	1.037 (0.958, 1.123)	0.366	1.195
Valine	1.018 (0.923, 1.122)	0.727	0.786	1.042 (0.952, 1.141)	0.374	0.510	0.955 (0.877, 1.040)	0.292	1.368
Citrate	1.031 (0.932, 1.141)	0.557	0.639	1.036 (0.943, 1.139)	0.463	0.585	0.926 (0.849, 1.008)	0.077	1.734
Glucose	1.065 (0.963, 1.177)	0.222	0.292	0.947 (0.861, 1.042)	0.262	0.390	1.089 (1.003, 1.184)	0.043	1.397
Lactate	0.971 (0.880, 1.071)	0.556	0.642	0.989 (0.902, 1.084)	0.809	0.854	1.034 (0.953, 1.122)	0.419	1.163
Glycoprotein acetyls, mainly a1-acid glycoprotein	0.994 (0.900, 1.098)	0.908	0.920	1.031 (0.940, 1.131)	0.518	0.630	0.901 (0.829, 0.979)	0.014	0.646
Acetoacetate	0.964 (0.875, 1.061)	0.452	0.541	0.917 (0.836, 1.007)	0.070	0.148	0.955 (0.879, 1.037)	0.273	1.708
Acetate	1.320 (1.197, 1.456)	<0.001	<0.001	1.198 (1.085, 1.323)	<0.001	0.006	1.310 (1.193, 1.439)	<0.001	<0.001
3-hydroxybutyrate	1.091 (0.987, 1.205)	0.089	0.134	1.005 (0.917, 1.101)	0.922	0.943	1.010 (0.928, 1.098)	0.826	0.962
Cholesterol esters to total lipids ratio in IDL	1.343 (1.215, 1.486)	<0.001	<0.001	1.289 (1.173, 1.418)	<0.001	<0.001	1.170 (1.070, 1.279)	0.001	0.062
Cholesterol esters to total lipids ratio in chylomicrons and extremely large VLDL	0.936 (0.848, 1.033)	0.189	0.255	1.245 (1.128, 1.374)	<0.001	0.001	1.052 (0.963, 1.149)	0.263	1.972
Cholesterol esters to total lipids ratio in large HDL	1.021 (0.922, 1.131)	0.687	0.754	0.958 (0.863, 1.064)	0.421	0.553	1.024 (0.942, 1.113)	0.580	1.011
Cholesterol esters to total lipids ratio in large LDL	1.214 (1.088, 1.356)	0.001	0.003	1.140 (1.031, 1.260)	0.011	0.046	1.090 (0.992, 1.198)	0.072	1.810
Cholesterol esters to total lipids ratio in large VLDL	1.025 (0.929, 1.131)	0.622	0.700	1.120 (1.020, 1.229)	0.017	0.061	0.962 (0.888, 1.043)	0.351	1.234
Cholesterol esters to total lipids ratio in medium HDL	1.104 (1.000, 1.218)	0.050	0.081	1.045 (0.961, 1.137)	0.304	0.432	1.020 (0.942, 1.104)	0.632	0.974
Cholesterol esters to total lipids ratio in medium LDL	0.809 (0.736, 0.889)	<0.001	<0.001	0.903 (0.822, 0.992)	0.033	0.094	0.958 (0.879, 1.044)	0.326	1.242
Cholesterol esters to total lipids ratio in medium VLDL	1.152 (0.977, 1.359)	0.093	0.139	1.112 (0.972, 1.273)	0.123	0.231	0.983 (0.919, 1.053)	0.628	0.982
Cholesterol esters to total lipids ratio in small HDL	1.029 (0.934, 1.134)	0.565	0.645	0.987 (0.913, 1.068)	0.754	0.815	1.033 (0.951, 1.122)	0.440	1.138
Cholesterol esters to total lipids ratio in small LDL	0.851 (0.772, 0.939)	0.001	0.004	1.010 (0.922, 1.107)	0.824	0.858	0.971 (0.897, 1.050)	0.458	1.107
Cholesterol esters to total lipids ratio in small VLDL	1.021 (0.931, 1.120)	0.657	0.728	1.052 (0.957, 1.156)	0.297	0.426	1.000 (0.925, 1.082)	0.996	1.005
Cholesterol esters to total lipids ratio in very large HDL	0.948 (0.858, 1.047)	0.293	0.364	0.931 (0.846, 1.024)	0.142	0.252	0.942 (0.864, 1.028)	0.180	2.131
Cholesterol esters to total lipids ratio in very large VLDL	1.020 (0.921, 1.130)	0.700	0.765	1.013 (0.926, 1.110)	0.772	0.831	0.983 (0.906, 1.067)	0.686	0.971
Cholesterol esters to total lipids ratio in very small VLDL	1.016 (0.927, 1.113)	0.740	0.790	1.040 (0.947, 1.142)	0.414	0.551	1.000 (0.924, 1.082)	0.997	0.997
Free cholesterol to total lipids ratio in IDL	0.991 (0.900, 1.092)	0.861	0.889	0.978 (0.892, 1.072)	0.630	0.723	0.990 (0.911, 1.076)	0.810	0.954
Free cholesterol to total lipids ratio in chylomicrons and extremely large VLDL	1.254 (1.136, 1.384)	<0.001	<0.001	1.192 (1.083, 1.312)	<0.001	0.006	1.068 (0.979, 1.164)	0.139	2.092
Free cholesterol to total lipids ratio in large HDL	0.992 (0.903, 1.089)	0.862	0.886	1.004 (0.922, 1.093)	0.934	0.951	1.026 (0.947, 1.112)	0.529	1.044
Free cholesterol to total lipids ratio in large LDL	0.886 (0.804, 0.976)	0.015	0.030	0.931 (0.847, 1.024)	0.143	0.251	0.952 (0.874, 1.037)	0.264	1.797
Free cholesterol to total lipids ratio in large VLDL	0.949 (0.857, 1.051)	0.314	0.389	0.967 (0.880, 1.063)	0.486	0.600	0.964 (0.892, 1.042)	0.350	1.251

Free cholesterol to total lipids ratio in medium HDL	1.076 (0.976, 1.187)	0.141	0.196	1.065 (0.980, 1.157)	0.135	0.243	1.033 (0.955, 1.119)	0.418	1.190
Free cholesterol to total lipids ratio in medium LDL	0.897 (0.813, 0.990)	0.031	0.055	0.950 (0.865, 1.043)	0.279	0.404	0.969 (0.900, 1.044)	0.413	1.223
Free cholesterol to total lipids ratio in medium VLDL	0.843 (0.762, 0.932)	0.001	0.003	0.870 (0.789, 0.959)	0.005	0.028	0.943 (0.869, 1.023)	0.160	2.253
Free cholesterol to total lipids ratio in small HDL	1.108 (1.002, 1.224)	0.046	0.075	1.073 (0.988, 1.164)	0.093	0.187	1.056 (0.973, 1.146)	0.193	2.173
Free cholesterol to total lipids ratio in small LDL	0.873 (0.794, 0.960)	0.005	0.011	0.949 (0.866, 1.040)	0.263	0.386	0.984 (0.905, 1.071)	0.715	0.975
Free cholesterol to total lipids ratio in small VLDL	0.879 (0.797, 0.969)	0.009	0.021	0.899 (0.819, 0.987)	0.025	0.080	0.986 (0.906, 1.073)	0.745	0.969
Free cholesterol to total lipids ratio in very large HDL	1.191 (1.075, 1.319)	0.001	0.003	1.091 (1.002, 1.187)	0.044	0.109	1.067 (0.983, 1.159)	0.123	2.124
Free cholesterol to total lipids ratio in very large VLDL	0.883 (0.800, 0.975)	0.014	0.028	0.940 (0.853, 1.037)	0.219	0.347	0.989 (0.906, 1.079)	0.807	0.955
Free cholesterol to total lipids ratio in very small VLDL	0.887 (0.805, 0.977)	0.015	0.031	0.883 (0.803, 0.971)	0.010	0.045	0.990 (0.910, 1.076)	0.806	0.964
Phospholipids to total lipds ratio in IDL	1.040 (0.942, 1.147)	0.438	0.528	0.946 (0.864, 1.037)	0.237	0.364	1.006 (0.926, 1.092)	0.889	0.986
Phospholipids to total lipds ratio in chylomicrons and extremely large VLDL	0.977 (0.893, 1.069)	0.608	0.687	0.985 (0.898, 1.079)	0.742	0.807	0.957 (0.879, 1.043)	0.316	1.246
Phospholipids to total lipds ratio in large HDL	1.032 (0.938, 1.135)	0.516	0.602	1.073 (0.986, 1.168)	0.104	0.202	1.021 (0.942, 1.107)	0.607	0.982
Phospholipids to total lipds ratio in large LDL	1.007 (0.903, 1.123)	0.900	0.916	0.976 (0.894, 1.065)	0.586	0.693	0.984 (0.913, 1.061)	0.674	0.972
Phospholipids to total lipds ratio in large VLDL	0.843 (0.763, 0.931)	0.001	0.003	0.900 (0.819, 0.990)	0.030	0.090	0.945 (0.871, 1.025)	0.171	2.137
Phospholipids to total lipds ratio in medium HDL	1.076 (0.976, 1.186)	0.141	0.197	1.065 (0.980, 1.157)	0.139	0.248	1.023 (0.946, 1.106)	0.571	1.011
Phospholipids to total lipds ratio in medium LDL	1.197 (1.082, 1.324)	<0.001	0.002	1.135 (1.032, 1.248)	0.009	0.041	1.029 (0.946, 1.118)	0.508	1.067
Phospholipids to total lipds ratio in medium VLDL	0.884 (0.804, 0.972)	0.011	0.023	0.932 (0.851, 1.021)	0.129	0.240	0.955 (0.879, 1.039)	0.285	1.426
Phospholipids to total lipds ratio in small HDL	1.161 (1.048, 1.288)	0.004	0.010	1.091 (1.003, 1.186)	0.041	0.107	1.048 (0.965, 1.137)	0.266	1.762
Phospholipids to total lipds ratio in small LDL	1.182 (1.070, 1.306)	0.001	0.003	0.955 (0.865, 1.053)	0.355	0.490	1.052 (0.971, 1.140)	0.212	2.270
Phospholipids to total lipds ratio in small VLDL	0.949 (0.863, 1.043)	0.276	0.351	0.967 (0.884, 1.058)	0.462	0.587	0.986 (0.906, 1.073)	0.738	0.965
Phospholipids to total lipds ratio in very large HDL	0.993 (0.905, 1.091)	0.889	0.909	0.960 (0.878, 1.048)	0.361	0.496	0.939 (0.865, 1.020)	0.137	2.194
Phospholipids to total lipds ratio in very large VLDL	0.893 (0.811, 0.983)	0.021	0.039	0.969 (0.884, 1.062)	0.499	0.611	0.973 (0.893, 1.061)	0.539	1.029
Phospholipids to total lipds ratio in very small VLDL	0.933 (0.849, 1.026)	0.151	0.207	0.947 (0.864, 1.037)	0.238	0.362	0.988 (0.908, 1.076)	0.786	0.972
Total cholesterol to total lipids ratio in IDL	0.824 (0.746, 0.910)	<0.001	0.001	0.839 (0.765, 0.921)	<0.001	0.005	0.905 (0.831, 0.985)	0.021	0.797
Total cholesterol to total lipids ratio in chylomicrons and extremely large VLDL	0.831 (0.755, 0.915)	<0.001	0.001	0.890 (0.810, 0.978)	0.015	0.056	0.961 (0.883, 1.047)	0.367	1.179
Total cholesterol to total lipids ratio in large HDL	1.028 (0.935, 1.131)	0.567	0.644	1.053 (0.967, 1.145)	0.234	0.360	1.022 (0.943, 1.108)	0.590	1.005
Total cholesterol to total lipids ratio in large LDL	0.997 (0.902, 1.102)	0.957	0.957	1.011 (0.923, 1.107)	0.816	0.858	0.969 (0.897, 1.047)	0.422	1.157
Total cholesterol to total lipids ratio in large VLDL	0.966 (0.876, 1.066)	0.495	0.580	1.056 (0.959, 1.162)	0.267	0.390	0.958 (0.885, 1.036)	0.282	1.478
Total cholesterol to total lipids ratio in medium HDL	1.111 (1.005, 1.227)	0.039	0.067	1.049 (0.965, 1.140)	0.262	0.388	1.025 (0.947, 1.110)	0.538	1.034
Total cholesterol to total lipids ratio in medium LDL	0.816 (0.743, 0.897)	<0.001	<0.001	0.902 (0.821, 0.991)	0.032	0.092	0.956 (0.878, 1.040)	0.291	1.393
Total cholesterol to total lipids ratio in medium VLDL	1.042 (0.940, 1.155)	0.438	0.530	1.073 (0.969, 1.188)	0.176	0.290	0.980 (0.909, 1.056)	0.590	0.998
Total cholesterol to total lipids ratio in small HDL	1.060 (0.959, 1.171)	0.252	0.326	1.012 (0.933, 1.096)	0.780	0.836	1.022 (0.942, 1.110)	0.599	0.991
Total cholesterol to total lipids ratio in small LDL	0.844 (0.767, 0.928)	<0.001	0.002	0.997 (0.910, 1.093)	0.956	0.969	0.971 (0.897, 1.051)	0.468	1.063
Total cholesterol to total lipids ratio in small VLDL	1.017 (0.926, 1.118)	0.723	0.785	1.049 (0.955, 1.151)	0.317	0.445	1.002 (0.922, 1.087)	0.971	1.011
Total cholesterol to total lipids ratio in very large HDL	1.017 (0.921, 1.124)	0.733	0.786	0.979 (0.894, 1.072)	0.644	0.735	0.973 (0.894, 1.058)	0.517	1.057
Total cholesterol to total lipids ratio in very large VLDL	1.037 (0.942, 1.142)	0.456	0.542	1.010 (0.922, 1.106)	0.833	0.864	1.005 (0.923, 1.093)	0.913	0.987
Total cholesterol to total lipids ratio in very small VLDL	1.016 (0.925, 1.116)	0.746	0.792	1.034 (0.942, 1.134)	0.485	0.603	1.001 (0.921, 1.088)	0.981	1.008
Triglycerides to total lipids ratio in IDL	0.995 (0.904, 1.096)	0.923	0.927	0.972 (0.888, 1.064)	0.539	0.648	0.993 (0.913, 1.080)	0.861	0.973
Triglycerides to total lipids ratio in chylomicrons and extremely large VLDL	1.216 (1.103, 1.341)	<0.001	0.001	1.166 (1.060, 1.283)	0.002	0.018	1.049 (0.963, 1.144)	0.274	1.623
Triglycerides to total lipids ratio in large HDL	1.012 (0.921, 1.113)	0.804	0.841	1.030 (0.946, 1.121)	0.495	0.608	1.027 (0.947, 1.114)	0.514	1.070
Triglycerides to total lipids ratio in large LDL	0.901 (0.816, 0.994)	0.037	0.065	0.929 (0.845, 1.022)	0.129	0.238	0.957 (0.880, 1.040)	0.297	1.309
Triglycerides to total lipids ratio in large VLDL	1.106 (1.004, 1.217)	0.041	0.069	1.000 (0.912, 1.096)	0.998	0.998	1.027 (0.944, 1.118)	0.532	1.040
Triglycerides to total lipids ratio in medium HDL	1.057 (0.959, 1.165)	0.262	0.337	1.036 (0.955, 1.124)	0.395	0.529	1.020 (0.943, 1.103)	0.616	0.990
Triglycerides to total lipids ratio in medium LDL	1.185 (1.069, 1.314)	0.001	0.004	0.999 (0.913, 1.093)	0.984	0.988	1.047 (0.959, 1.142)	0.305	1.295
Triglycerides to total lipids ratio in medium VLDL	1.079 (0.979, 1.190)	0.124	0.176	0.965 (0.879, 1.060)	0.461	0.589	1.015 (0.932, 1.105)	0.730	0.966
Triglycerides to total lipids ratio in small HDL	0.970 (0.881, 1.069)	0.543	0.629	0.977 (0.896, 1.065)	0.595	0.701	1.078 (0.993, 1.170)	0.072	2.017
Triglycerides to total lipids ratio in small LDL	1.163 (1.059, 1.277)	0.002	0.005	1.060 (0.969, 1.159)	0.205	0.330	0.999 (0.919, 1.087)	0.987	1.000
Triglycerides to total lipids ratio in small VLDL	1.075 (0.977, 1.184)	0.139	0.196	0.984 (0.896, 1.079)	0.727	0.797	1.033 (0.950, 1.123)	0.450	1.112
Triglycerides to total lipids ratio in very large HDL	0.989 (0.895, 1.092)	0.820	0.850	1.017 (0.927, 1.116)	0.717	0.791	1.035 (0.951, 1.126)	0.429	1.136
Triglycerides to total lipids ratio in very large VLDL	1.171 (1.065, 1.287)	0.001	0.004	1.066 (0.975, 1.165)	0.158	0.268	1.016 (0.935, 1.104)	0.706	0.968
Triglycerides to total lipids ratio in very small VLDL	1.106 (1.007, 1.215)	0.035	0.061	1.044 (0.955, 1.141)	0.341	0.476	1.042 (0.958, 1.133)	0.335	1.236
Total cholines	1.065 (0.967, 1.173)	0.201	0.269	1.054 (0.964, 1.152)	0.249	0.376	1.035 (0.952, 1.127)	0.418	1.177
Phosphatidylcholine and other cholines	1.172 (1.065, 1.289)	0.001	0.004	1.068 (0.978, 1.166)	0.143	0.250	1.052 (0.967, 1.145)	0.238	2.229
Total phosphoglycerides	0.928 (0.845, 1.020)	0.121	0.173	0.966 (0.887, 1.051)	0.421	0.551	1.036 (0.957, 1.122)	0.385	1.203
Ratio of triglycerides to phosphoglycerides	1.085 (0.986, 1.194)	0.096	0.142	1.036 (0.947, 1.132)	0.442	0.568	1.022 (0.939, 1.112)	0.619	0.981
Albumin	1.160 (1.055, 1.274)	0.002	0.006	1.137 (1.041, 1.242)	0.004	0.026	1.047 (0.963, 1.139)	0.284	1.451
Creatinine	0.871 (0.789, 0.961)	0.006	0.013	0.913 (0.833, 1.002)	0.055	0.130	0.870 (0.799, 0.947)	0.001	0.097
Estimated degree of unsaturation	1.228 (1.082, 1.395)	0.002	0.005	0.971 (0.852, 1.106)	0.656	0.742	1.176 (1.063, 1.301)	0.002	0.093
Sphingomyelins	0.903 (0.823, 0.991)	0.031	0.055	1.019 (0.929, 1.117)	0.694	0.769	0.924 (0.843, 1.013)	0.092	1.890

Models were adjusted for age, sex, region, education, household income, occupation, marital status, tea drinking habit, smoking status, alcohol intake, physical activity, self-rated health, fasting time, and frequency of 12 food groups.
