# Supplementary material for: Association of Red Meat Consumption, Metabolic Markers, and Risk of Cardiovascular Diseases
Source: Front Nutr. 2022 Apr 15;9:833271. doi: 10.3389/fnut.2022.833271 (PMC9051033; doi:10.3389/fnut.2022.833271)
Supplement: Supplementary file 4 [file Data_Sheet_1.pdf]

Supplementary Material

1 Supplementary Data

1.1 Food frequency questionnaire (FFQ) at baseline.

During the past 12 months, about how often did you eat the following foods?

	Daily	4-6 days per week	1-3 days per week	Monthly	Never/rarely
Rice	☐	☐	☐	☐	☐
Wheat	☐	☐	☐	☐	☐
Other staple food (corn, millet etc.)	☐	☐	☐	☐	☐
Meat	☐	☐	☐	☐	☐
Poultry	☐	☐	☐	☐	☐
Fish/sea food	☐	☐	☐	☐	☐
Fresh eggs	☐	☐	☐	☐	☐
Fresh vegetables	☐	☐	☐	☐	☐
Soybean products	☐	☐	☐	☐	☐
Preserved vegetables	☐	☐	☐	☐	☐
Fresh fruit	☐	☐	☐	☐	☐
Dairy products (milk, yogurt)	☐	☐	☐	☐	☐

1.2 Food frequency questionnaire (FFQ) in the 2nd re-survey.

During the past 12 months, about how often and how much did you eat the following foods?

	Daily	4-6 days per week	1-3 days per week	Monthly	Never/rarely	Amount on consuming days
Rice	☐	☐	☐	☐	☐	____×50g
Wheat products	☐	☐	☐	☐	☐	____×50g
Other staple foods	☐	☐	☐	☐	☐	____×50g
Meat	☐	☐	☐	☐	☐	____×50g
Poultry	☐	☐	☐	☐	☐	____×50g
Fish/seafood	☐	☐	☐	☐	☐	____×50g
Eggs	☐	☐	☐	☐	☐	____egg(s)
Fresh vegetables	☐	☐	☐	☐	☐	____×50g
Fresh fruit	☐	☐	☐	☐	☐	____×100g
Soya products (excluding liquids) *	☐	☐	☐	☐	☐	____×50g
Soymilk *	☐	☐	☐	☐	☐	____ml
Salted vegetables *	☐	☐	☐	☐	☐	____×50g
Pickled vegetables *	☐	☐	☐	☐	☐	____×50g
Milk *	☐	☐	☐	☐	☐	____ml
Yoghurt *	☐	☐	☐	☐	☐	____×50g
Other dairy foods *	☐	☐	☐	☐	☐	____g
Dried vegetables **	☐	☐	☐	☐	☐	____×50g
Pure fruit/vegetable juice **	☐	☐	☐	☐	☐	____ml
Carbonated soft drinks **	☐	☐	☐	☐	☐	____ml
Other cold soft drinks **	☐	☐	☐	☐	☐	____ml

* Split food groups; ** Added food groups.

1.3 24 hours dietary recall (24-HDR) table that used for validation.

Have you eaten the same food in the last 24 hours as you normally do? ☐ Yes ☐ No

Food	Raw material	Raw material weight	Edible weight	Meal time	Meal place	Production time	Production place

1.4 Members of the China Kadoorie Biobank collaborative group.

International Steering Committee: Junshi Chen, Zhengming Chen (PI), Robert Clarke, Rory Collins, Yu Guo, Liming Li (PI), Jun Lv, Richard Peto, Robin Walters. **International Co-ordinating Centre, Oxford:** Daniel Avery, Ruth Boxall, Derrick Bennett, Yumei Chang, Yiping Chen, Zhengming Chen, Robert Clarke, Huaidong Du, Simon Gilbert, Alex Hacker, Mike Hill, Michael Holmes, Andri Iona, Christiana Kartsonaki, Rene Kerosi, Ling Kong, Om Kurmi, Garry Lancaster, Sarah Lewington, Kuang Lin, John McDonnell, Iona Millwood, Qunhua Nie, Jayakrishnan Radhakrishnan, Paul Ryder, Sam Sansome, Dan Schmidt, Paul Sherliker, Rajani Sohoni, Becky Stevens, Iain Turnbull, Robin Walters, Jenny Wang, Lin Wang, Neil Wright, Ling Yang, Xiaoming Yang. **National Co-ordinating Centre, Beijing:** Yu Guo, Xiao Han, Can Hou, Jun Lv, Pei Pei, Chao Liu, Canqing Yu. **10 Regional Co-ordinating Centres: Qingdao CDC:** Zengchang Pang, Ruqin Gao, Shanpeng Li, Shaojie Wang, Yongmei Liu, Ranran Du, Yajing Zang, Liang Cheng, Xiaocao Tian, Hua Zhang, Yaoming Zhai, Feng Ning, Xiaohui Sun, Feifei Li. **Licang CDC:** Silu Lv, Junzheng Wang, Wei Hou. **Heilongjiang Provincial CDC:** Mingyuan Zeng, Ge Jiang, Xue Zhou. **Nangang CDC:** Liqiu Yang, Hui He, Bo Yu, Yanjie Li, Qinai Xu,Quan Kang, Ziyan Guo. **Hainan Provincial CDC:** Dan Wang, Ximin Hu, Jinyan Chen, Yan Fu, Zhenwang Fu, Xiaohuan Wang. **Meilan CDC:** Min Weng, Zhendong Guo, Shukuan Wu,Yilei Li, Huimei Li, Zhifang Fu. **Jiangsu Provincial CDC:** Ming Wu, Yonglin Zhou, Jinyi Zhou, Ran Tao, Jie Yang, Jian Su. **Suzhou CDC:** Fang liu, Jun Zhang, Yihe Hu, Yan Lu, , Liangcai Ma, Aiyu Tang, Shuo Zhang, Jianrong Jin, Jingchao Liu. **Guangxi Provincial CDC:** Zhenzhu Tang, Naying Chen, Ying Huang. **Liuzhou CDC:** Mingqiang Li, Jinhuai Meng, Rong Pan, Qilian Jiang, Jian Lan,Yun Liu, Liuping Wei, Liyuan Zhou, Ningyu Chen Ping Wang, Fanwen Meng, Yulu Qin,, Sisi Wang. **Sichuan Provincial CDC:** Xianping Wu, Ningmei Zhang, Xiaofang Chen,Weiwei Zhou. **Pengzhou CDC:** Guojin Luo, Jianguo Li, Xiaofang Chen, Xunfu Zhong, Jiaqiu Liu, Qiang Sun. **Gansu Provincial CDC:** Pengfei Ge, Xiaolan Ren, Caixia Dong. **Maiji CDC:** Hui Zhang, Enke Mao, Xiaoping Wang, Tao Wang, Xi zhang. **Henan Provincial CDC:** Ding Zhang, Gang Zhou, Shixian Feng, Liang Chang, Lei Fan. **Huixian CDC:** Yulian Gao, Tianyou He, Huarong Sun, Pan He, Chen Hu, Xukui Zhang, Huifang Wu, Pan He. **Zhejiang Provincial CDC:** Min Yu, Ruying Hu, Hao Wang. Tongxiang CDC: Yijian Qian, Chunmei Wang, Kaixu Xie, Lingli Chen, Yidan Zhang, Dongxia Pan, Qijun Gu. **Hunan Provincial CDC:** Yuelong Huang, Biyun Chen, Li Yin, Huilin Liu, Zhongxi Fu, Qiaohua Xu. **Liuyang CDC:** Xin Xu, Hao Zhang, Huajun Long, Xianzhi Li, Libo Zhang, Zhe Qiu.

2 Supplementary Figures and Tables

2.1 Supplementary Figures

2.1.1 Figure S1

The flowchart that summarized the data analysis and integration. The CKB is a prospective cohort of 512,725 adults. The present study included 4,778 participants, who had metabolomics data, from a previous nested case-control study based on CKB. Cases consisted of 946, 1,217, and 1,238 incident MI, IS, and ICH cases, respectively, with a censoring date of 1 January 2015. The incident cases were identified through electronic linkage via a unique personal identification number to established death registries and the universal nationwide health insurance system. A total of 1,377 controls were frequently matched to the combined cases by age, sex, and area if possible. All cases and controls had no history of self-reported prior doctor-diagnosed coronary heart disease (CHD), transient ischemic attack (TIA), stroke, and cancer, and were not using statin therapy at baseline. Habitual frequency of red meat during the past 12 months was asked using the interviewer-administered, laptop-based food frequency questionnaire (FFQ) at baseline. Baseline plasma samples were undergone high-throughput targeted NMR spectroscopy to quantify 225 absolute concentrations of metabolic markers or derived traits. Measurements below the limit of detection (LOD) for each metabolite were imputed with the lowest measured concentration. Each metabolic marker was log-transformed and divided by its standard deviation (SD). Linear regression was conducted to evaluate the effects of red meat consumption on metabolic markers, which were further compared with the effects of these markers on CVD risk assessed by logistic regression. We also performed several sensitivity analyses to examine the robustness of associations between red meat consumption and metabolic markers.

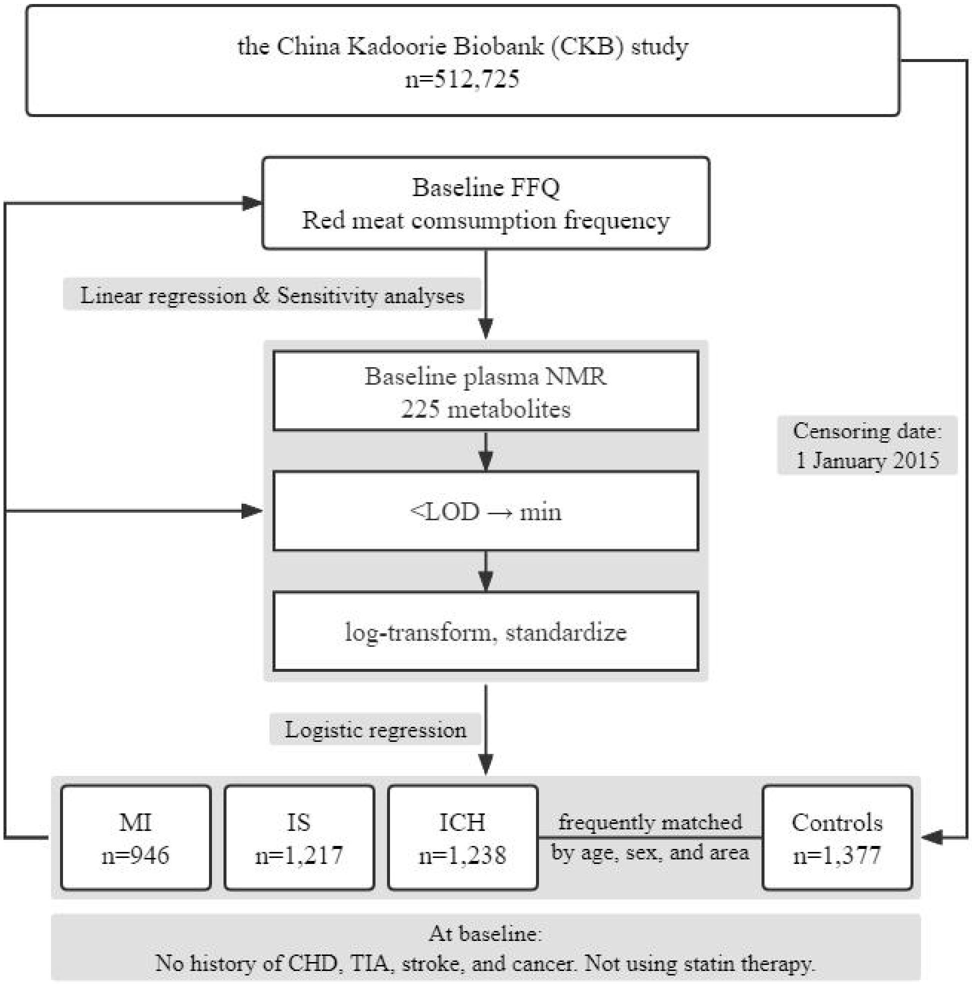
the China Kadoorie Biobank (CKB) study
n=512,725
Baseline FFQ
Red meat comsumption frequency
Linear regression & Sensitivity analyses
Baseline plasma NMR
225 metabolites
<LOD → min
log-transform, standardize
Censoring date:
1 January 2015
Logistic regression
MI
n=946
IS
n=1,217
ICH
n=1,238
frequently matched
by age, sex, and area
Controls
n=1,377
At baseline:
No history of CHD, TIA, stroke, and cancer. Not using statin therapy.

2.1.2 Figure S2

Associations of red meat consumption and log-transformed metabolic markers (A) in basic models, additionally adjusted for (B) BMI, (C) hypertension, (D) diabetes, and (E) family history of diabetes, heart attack, or stroke. Significance: *p<0.05, **p<0.01, ***p<0.001 (FDR-adjusted p using the Benjamini-Hochberg method).

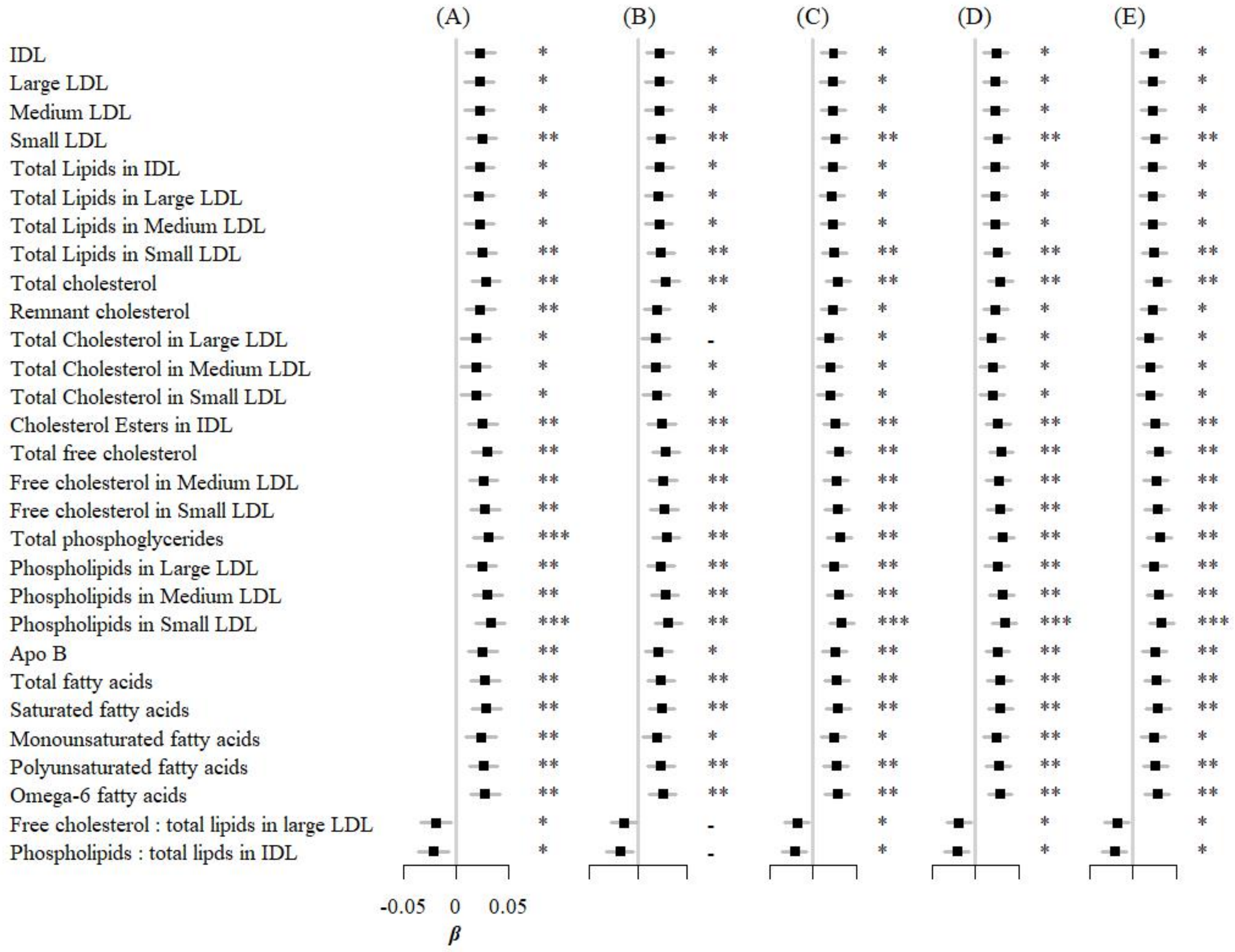

2.1.3 Figure S3

Associations of red meat consumption and log-transformed metabolic markers (A) in basic models, when (B) restricting the analyses to participants without any metabolic markers below the detection limit or rejected by the quality control (n=4,251), and (C) using the weekly amount of red meat consumption as a continuous independent variable instead of its frequency. Significance: *p<0.05, **p<0.01, ***p<0.001 (FDR-adjusted p using the Benjamini-Hochberg method).

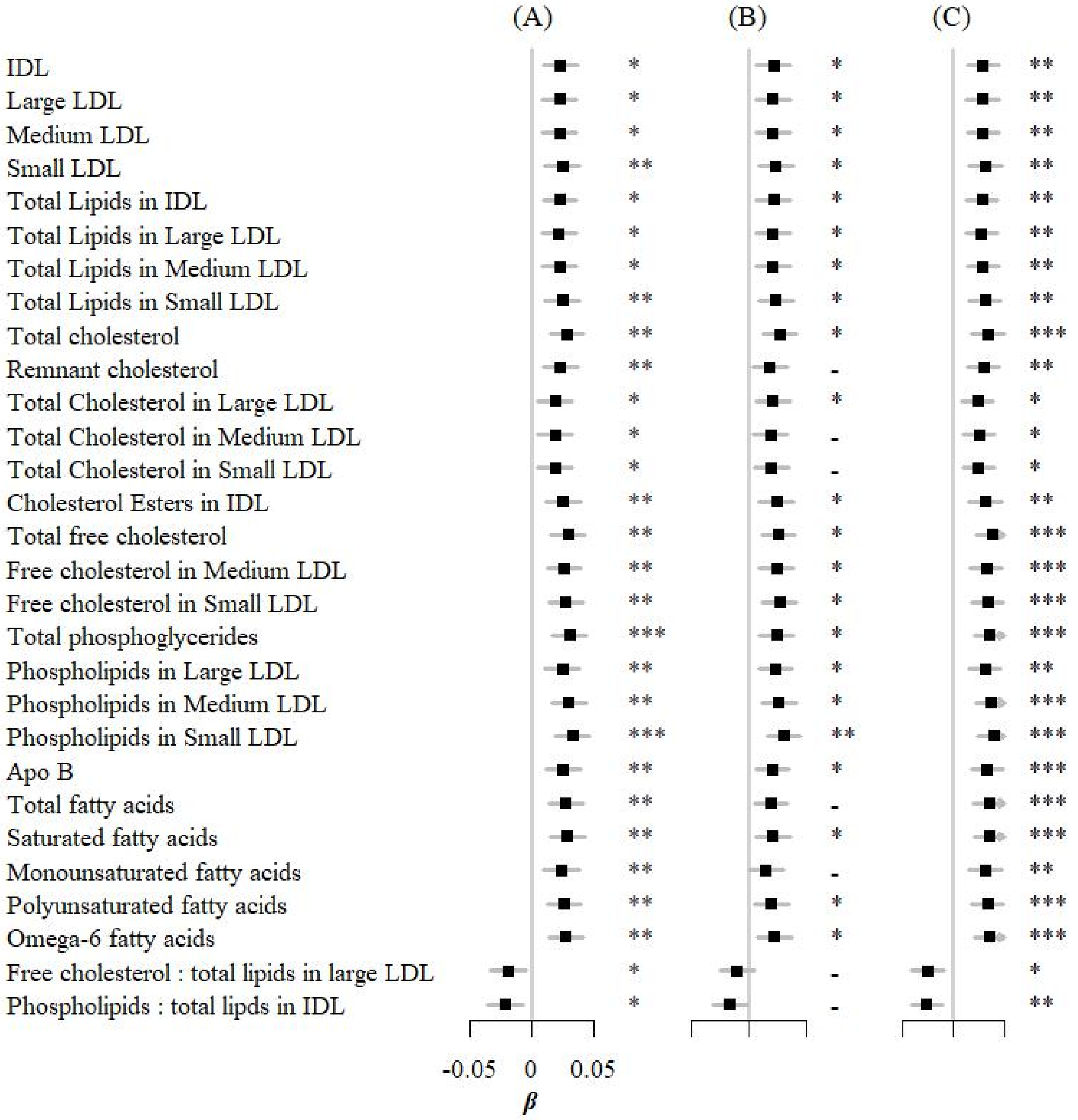

2.2 Supplementary Tables

Since the supplementary tables (Table S1~S3) were too wide to fit typesetting, we uploaded three PDF files separately.
